# Galectin-3, a novel endogenous TREM2 ligand, detrimentally regulates inflammatory response in Alzheimer’s disease

**DOI:** 10.1007/s00401-019-02013-z

**Published:** 2019-04-20

**Authors:** Antonio Boza-Serrano, Rocío Ruiz, Raquel Sanchez-Varo, Juan García-Revilla, Yiyi Yang, Itzia Jimenez-Ferrer, Agnes Paulus, Malin Wennström, Anna Vilalta, David Allendorf, Jose Carlos Davila, John Stegmayr, Sebastian Jiménez, Maria A. Roca-Ceballos, Victoria Navarro-Garrido, Maria Swanberg, Christine L. Hsieh, Luis M. Real, Elisabet Englund, Sara Linse, Hakon Leffler, Ulf J. Nilsson, Guy C. Brown, Antonia Gutierrez, Javier Vitorica, Jose Luis Venero, Tomas Deierborg

**Affiliations:** 1 Department of Experimental Medical Science, Experimental Neuroinflammation Laboratory, Lund University, 221 84 Lund, Sweden; 2 Departamento de Bioquímica y Biología Molecular, Instituto de Biomedicina de Sevilla (IBiS, HUVR/CSIC/Universidad de Sevilla), Universidad de Sevilla, Seville, Spain; 3 Departamento de Biología Celular, Genética y Fisiología, Instituto de Investigación Biomédica de Málaga (IBIMA), Facultad de Ciencias, Universidad de Málaga, Málaga, Spain; 4 Department of Biochemistry, University of Cambridge, Cambridge, UK; 5 Clinical Memory Research Unit, Department of Clinical Sciences Malmö, Lund University, Malmö, Sweden; 6 Department of Biochemistry and Structural Biology, Lund University, Lund, Sweden; 7 Unidad Clínica de Enfermedades Infecciosas y Microbiología, Hospital Universitario de Valme, Seville, Spain; 8 Division of Oncology and Pathology, Department of Clinical Sciences, Lund University, Lund, Sweden; 9 Department of Laboratory Medicine, Division of Microbiology, Immunology and Glycobiology (MIG), Lund University, Lund, Sweden; 10 Department of Chemistry, Centre for Analysis and Synthesis, Lund University, Lund, Sweden; 11 Translational Neurogenetics Unit, Department of Experimental Medical Science, Wallenberg Neuroscience Center, Lund University, 221 84 Lund, Sweden; 12 Immunology Section, Department of Medicine, San Francisco VA Medical Center, UCSF School of Medicine, 4150 Clement St. 111R, San Francisco, CA 94121 USA; 13 Centro de Investigación Biomédica en Red sobre Enfermedades Neurodegenerativas (CIBERNED), Madrid, Spain

**Keywords:** Alzheimer’s disease (AD), Galectin-3, TREM2, Microglia, Inflammation, Amyloid aggregation

## Abstract

Alzheimer’s disease (AD) is a progressive neurodegenerative disease in which the formation of extracellular aggregates of amyloid beta (Aβ) peptide, fibrillary tangles of intraneuronal tau and microglial activation are major pathological hallmarks. One of the key molecules involved in microglial activation is galectin-3 (gal3), and we demonstrate here for the first time a key role of gal3 in AD pathology. Gal3 was highly upregulated in the brains of AD patients and 5xFAD (familial Alzheimer’s disease) mice and found specifically expressed in microglia associated with Aβ plaques. Single-nucleotide polymorphisms in the *LGALS3* gene, which encodes gal3, were associated with an increased risk of AD. Gal3 deletion in 5xFAD mice attenuated microglia-associated immune responses, particularly those associated with TLR and TREM2/DAP12 signaling. In vitro data revealed that gal3 was required to fully activate microglia in response to fibrillar Aβ. Gal3 deletion decreased the Aβ burden in 5xFAD mice and improved cognitive behavior. Interestingly, a single intrahippocampal injection of gal3 along with Aβ monomers in WT mice was sufficient to induce the formation of long-lasting (2 months) insoluble Aβ aggregates, which were absent when gal3 was lacking. High-resolution microscopy (stochastic optical reconstruction microscopy) demonstrated close colocalization of gal3 and TREM2 in microglial processes, and a direct interaction was shown by a fluorescence anisotropy assay involving the gal3 carbohydrate recognition domain. Furthermore, gal3 was shown to stimulate TREM2–DAP12 signaling in a reporter cell line. Overall, our data support the view that gal3 inhibition may be a potential pharmacological approach to counteract AD.

**Electronic supplementary material:**

The online version of this article (10.1007/s00401-019-02013-z) contains supplementary material, which is available to authorized users.

## Introduction

Alzheimer’s disease (AD) is the leading cause of dementia, affecting more than 24 million people worldwide [4] and with the incidence of AD increasing dramatically as the global population ages. The classical hallmarks of AD include the formation of amyloid-beta (Aβ) plaque deposits and neurofibrillary tangles (NFT) containing abnormal hyperphosphorylation of tau. Over the course of the disease, the deposition of Aβ and the formation of the NFTs appear to be correlated with the on-going hippocampal neurodegeneration [6]. Hippocampal neurodegeneration has a negative effect on the cognitive ability of patients, especially in the formation of new memories. The mechanisms triggering the deposition of the Aβ or the formation of NFTs are currently under investigation. However, several mechanisms and factors have been suggested to be involved in the initiation and the progression of the disease, including activation of the innate immune system, environmental factors and lifestyle [42]. The innate immune system has been widely studied and has been implicated in several neurodegenerative diseases [19]. Over the last few years, several studies have suggested that inflammation plays a major role in the initiation and progression of AD [17, 20, 62, 66].

The inflammatory process in the central nervous system (CNS) is generally referred to as neuroinflammation. Glial cells have a leading role in propagating neuroinflammation. Among glial cells, microglia are considered the main source of proinflammatory molecules within the brain [18, 27, 63]. It is believed that sustained release of proinflammatory molecules such as cytokines, chemokines, nitrogen reactive species (NRS) or reactive oxygen species (ROS) can create a neurotoxic environment that drives the progression of AD [1, 19, 46]. Moreover, there is strong evidence supporting the role of inflammatory molecules, such as iNOS, in the initiation of plaque formation due to post-translational modifications of Αβ, leading to a faster aggregation [31, 32]. Indeed, counteracting the aggregation process has been proposed as a therapeutic strategy to alleviate the progression of the pathology [58].

Conclusive evidence supporting a direct role of microglia in human neurodegeneration was revealed with the recent advent of massive genome analysis. Specifically, genetic-associated studies have identified several AD-risk genes strongly associated with the innate immune system, including, among others, triggering receptor on myeloid cells 2 (*trem2*), *cd33*, *cr1*, *clu*, *epha1* and *ms4a4a/ms4a6a* [7, 16]. Recent single-cell transcriptomic studies of microglia have pointed out galectin-3 (Lgals3; gal3) as one of the most attractive molecules in brain innate immunity associated with neurodegeneration (see recent review [10]). Indeed, Holtman et al. [22] analyzed transcriptional profiles of isolated microglia from different disease mouse models, including AD, amyotrophic lateral sclerosis and aging, and revealed a shared transcriptional network in all conditions, including a strong upregulation of gal3. Interestingly, the authors analyzed the most likely candidates to orchestrate the microglial activation phenotype and found four major hub genes: *Lgals3*, *Igf1*, *Csf1* and *Axl* [22]. Two other recent studies characterized the molecular signature of microglial cells associated with different disease conditions, including aging and AD [27, 30]. Again, a common microglial neurodegenerative disease-associated phenotype was identified, supporting that (1) the microglial phenotype was driven by TREM2 and (2) gal3 was one of the most upregulated genes.

Recent findings from our group have demonstrated that gal3 is released by LPS-treated microglia and acts as a ligand of toll-like receptor 4 (TLR4), which is one of the canonical receptors involved in the microglial inflammatory response [9]. Based on our previous studies and recent findings from the AD field related to the role of inflammation in the disease, we hypothesize that the reduction of microglial activation by removing/inhibiting gal3 will counteract the inflammatory response present in AD and slow the progression of the disease. In this work, we demonstrate (1) a significant upregulation of gal3 in human AD patients compared to age-matched healthy controls, (2) the preferential expression of gal3 in microglial cells in contact with Aβ plaques both in human and mouse, (3) a clear reduction of the inflammatory response in microglial cells challenged with Aβ fibrils (fAβ) following gal3 inhibition and gal3 deletion in microglial cultures, (4) a significant Aβ plaque reduction and better cognitive outcome in 5xFAD mice lacking gal3, and (5) the role of gal3 as a ligand of TREM2 through its carbohydrate recognition domain (CRD) domain, which confers the ability to bind glycans.

## Materials and methods

### Animals

5xFAD–Gal3 KO transgenic mice were generated by crossing heterozygous 5xFAD (±) mice with homozygous Gal3KO (−/−) mice to get 5xFAD (±)/Gal3 (±) mice. Subsequent crossings between animals expressing this genotype allowed for the generation of 5xFAD (±)–Gal3 (−/−) mice, hence referred to as 5xFAD/Gal3KO mice. 5xFAD/Gal3 mice are hence referred to as 5xFAD mice. Male and female mice were equally distributed between the experimental groups. No differences were found between male and female mice in any of the experiments performed. All animal experiments were performed in accordance with the animal research regulations (RD53/2013 and 2010/63/UE) in Spain and European Union and with the approval of the Committee of Animal Research at the University of Seville (Spain).

For primary microglial cultures, Gal3KO mice with a C57BL/6 background were obtained from Dr. K. Sävman at Gothenburg University. All procedures were carried out in accordance with the international guidelines on experimental animal research and were approved by the Malmö-Lund Ethical Committee for Animal Research in Sweden (M250-11, M30-16, Dnr 5.8.18-01107/2018).

### Genotyping

The genotypes of Gal3−/− (KO) and Gal3+/+ (WT) mice were determined using an integrated extraction and amplification kit (Extract-N-Amp™, Sigma-Aldrich). First, the samples were incubated at 94 °C for 5 min, followed by 40 cycles with denaturation at 94 °C for 45 s, annealing at 55 °C for 30 s, and elongation at 72 °C for 1.5 min. The following primers (CyberGene, Solna, Sweden) were used: galectin-3 common (5′-CAC GAA CGT CTT TTG CTC TCT GG-3′), galectin-3−/− (5′-GCT TTT CTG GAT TCA TCG ACT GTG G-3′, single band of 384 bp) and galectin-3+/+ (5′-TGA AAT ACT TAC CGA AAA GCT GTC TGC-3′, single band of 300 bp) [17]. For the 5xFAD mice, the primers (5′–3′) used are listed below: APP Forward AGGACTGACCACTCGACCAG, APP Reverse CGGGGGTCTAGTTCTGCAT, PSN1 Forward AATAGAGAACGGCAGGAGCA, PSN1 Reverse GCCATGAGGGCACTAATCAT, WT APP Forward CTAGGCCACAGAATTGAAAGATCT, WTT APP Reverse GTAGGTGGAAATTCTAGCATCATCC, RD1, RD2 and RD3 AAGCTAGCTGCAGTAACGCCATTT ACCTGCATGTGAACCCAGTATTCTATC, CTACAGCCCCTCTCCAAGGTTTATAG. The PCR products were labeled with SYBR^®^ Green (Sigma-Aldrich), separated by gel electrophoresis and visualized using a CCD camera (SONY, Tokyo, Japan).

### Protein preparation

Aβ (M1–42) (i.e., with a starting Met0 to allow for production of otherwise untagged peptide) was expressed in *E. coli* (BL21 DE3 PLysS Star) and purified from inclusion bodies after repeated sonication using ion exchange in batch mode and size exclusion chromatography in column format. This was followed by lyophylization of monomer aliquots. Each day, before use in any experiment, Aβ42 monomer was again isolated from the aliquots by dissolving an aliquot in 1 mL of 6 M GuHCl, and, using gel filtration (Superdex 75, 10–300 column) with 20 mM sodium phosphate, 0.2 mM EDTA, pH 8.0 as running buffer, collected on ice in a low-binding tube (Genuine Axygen Quality, Microtubes, MCT-200-L-C, Union City, CA, USA). The concentration was determined by absorbance at 280 nm using *ε*_280_ = 1440 M^−1^ cm^−1^. The solution was diluted with buffer and supplemented with concentrated NaCl to achieve 10 µM monomer and 150 mM NaCl. The solution was placed in wells of a PEGylated polystyrene plate (Corning 3881) and sealed with a plastic film to avoid evaporation. Thioflavin T (ThT) was added to one well from a concentrated stock to obtain 6 µM ThT. Plates were incubated at 37 °C in a FLUOstar Omega plate reader under quiescent conditions (BMG Labtech, Offenburg, Germany), and the fibril formation of amyloid-beta was followed by reading the fluorescence (excitation 440 nm, emission 480 nm) through the bottom of the plate. The samples in wells without ThT were collected after reaching the plateau of the sigmoidal transition (after ca. 1 h).

### Cryogenic transmission electron microscopy (cryo-TEM)

Specimens for electron microscopy were prepared in a controlled environment vitrification system (CEVS) to ensure stable temperature and to avoid the loss of solution during sample preparation. The specimens were prepared as thin liquid films, < 300 nm thick, on lacey carbon-filmed copper grids and plunged into liquid ethane at − 180 °C. This led to vitrified specimens, avoiding component segmentation and rearrangement and water crystallization, thereby preserving the original microstructures. The vitrified specimens were stored under liquid nitrogen until measured. An Oxford CT3500 cryoholder and its workstation were used to transfer the specimen into the electron microscope (Philips CM120 BioTWIN Cryo) equipped with a post-column energy filter (Gatan GIF100). The acceleration voltage was 120 kV. The images were recorded digitally with a CCD camera under low electron-dose conditions. The node-to-node distance was measured using the software Digital Graph (Gatan Inc.).

### Endotoxin test

To further evaluate the properties of our protein preparation, we performed an endotoxin assay to ensure that BV2 microglial cell activation was not due to the presence of endotoxins such as LPS (suppl. Fig. 5, online resource 5). Fibril preparations used in the in vitro and in vivo experiments were tested for endotoxins using the Pierce^®^ LAL Chromogenic Endotoxin Quantitation Kit, (ThermoScientific) according to the manufacturer’s instructions (suppl. Fig. 5a, online resource 5).

### XTT (cell viability) assay

XTT assay was performed to measure mitochondrial activity (mitochondrial dehydrogenase) in living cells using XTT (2,3-bis-(2-methoxy-4-nitro-5-sulfophenyl)-2*H*-tetrazolium-5-carboxyanilide salt) (Sigma-Aldrich, Sweden). The assay was performed following the manufacturer’s protocol in a 96-well plate (Biochrom Asys Expert 96 micro plate reader, Cambridge, UK) (suppl. Fig. 5c, online resource 5).

### Cell lines and primary cultures

BV2 microglial cells were cultured in 12-well plates, 250,000 cells/well and stimulated with Aβ monomers and fibrils for a range of time points (3, 6, 12 and 24 h) and concentrations (3 and 10 μM). Primary microglial cells were obtained from WT and Gal3KO mice. The primary cells were obtained from the cortex, as previously described [15], and cultured for 14 days in T75 flask culture conditions before treatment. After 14 days, microglial cells were isolated, and 25,000 were cultured in 96-well plates and incubated for 12 h with fAβ. Cell viability and number of cells were assessed using a TC20 Bio-Rad Cell counter, Bio-Rad chambers slides and trypan blue 0.04% for BV2 and primary cultures. The same number of viable cells of each genotype (WT or Gal3KO) was plated per well. The cells were grown in DMEM (Invitrogen), FBS (Invitrogen) 10% (v/v) and penicillin–streptomycin (Invitrogen) 1% (v/v) in 5% CO_2_ in air at 37 °C in a humidified incubator. After incubation, the media was collected to measure extracellular cytokines and proteins to evaluate the microglial activation profile.

### TREM2–DAP12 reporter cell line

The ability of gal3 to activate TREM2–DAP12 signaling was assayed in a BWZ thymoma reporter cell line transfected with TREM2 and DAP12 as previously described [23]. In these cells, TREM2/DAP12 signaling activates phospholipase C, leading to a calcium influx that activates calcineurin, which, in turn, leads to disinhibition of the nuclear import of NFAT (nuclear factor of activated T-cells). This, in turn, induces transcription of the LacZ β-galactosidase gene. The TREM2/DAP12 reporter cells or parental BWZ cells not expressing TREM2 or DAP12 (control) were incubated with the indicated concentrations of gal3 for 24 h at 37 °C. Afterwards, they were washed and then lysed in a buffer containing 100 mM 2-ME, 9 mM MgCl_2_, 0.125% NP-40, and 30 mM chlorophenol red galactosidase (CPRG). Plates were developed for 24 h at 37 °C, and lacZ activity was measured as previously described [23]. As a positive control, ionomycin (3 μM) was added.

### Phagocytosis experiments

After 24 h of primary microglia culturing, 1 µM of gal3 was added for 30 min prior to incubation or simultaneously with labeled Aβ1-42 (HiLyte™ Fluor 647-labeled, Human; ANA64161) as monomers (mAβ) or fibrils (fAβ) for 1 h. fAβ samples were obtained by incubating them for 24 h at 37 °C. Cells were detached by brief incubation with trypsin with EDTA and analyzed by flow cytometry (Accuri C6, BD Biosciences). Mean fluorescence in the FL4 gate was used to plot results and is expressed as a percentage from Aβ1–42 uptake into primary microglia.

### Sequential protein extraction

Soluble and insoluble protein fractions were obtained from the whole cortex (mice) and temporal cortex (human) using sequential protein extraction. For each sample, fractions were obtained by homogenization of the cortex with a Dounce homogenizer in the presence of PBS (1 mL/100 µg of tissue). The supernatant, the S1 fraction, was aliquoted and stored at − 80 °C. The S1 soluble fraction was obtained after centrifugation for 1 h at 40,000 rpm in special tubes for high-speed centrifugation by Beckman-Coulter. The pellet was dissolved in RIPA buffer (Sigma-Aldrich, Germany) and subsequently ultracentrifuged at 30,000 rpm. The resulting supernatant, the S2 fraction (intracellular particulate proteins), was aliquoted and stored. The pellet was re-dissolved in buffered-SDS (2% SDS in 20 mM Tris–HCl, pH 7.4, 140 mM NaCl) and then centrifuged as above. The supernatant, the S3 fraction (SDS releasable proteins), was stored. Finally, the remaining pellet (P3) was dissolved in SDS–urea (20 mM Tris–HCl, pH 7.4, 4% SDS and 8 M urea). PBS and RIPA solution were prepared with a protein inhibitor (Protein Inhibitor Cocktail, ThermoScientific) to prevent protein degradation and to inhibit the enzymatic activity of phosphatases (PhosphoStop, Roche).

### Western blotting

Proteins were extracted from cell cultures with RIPA buffer (Sigma-Aldrich, Germany) along with proteinase and phosphatase inhibitors (Roche, Switzerland). Protein concentration was measured using a BCA kit according to the manufacturer’s protocol (BCA Protein Assay-Kit, ThermoScientific, Sweden). Protein extracts were then separated by SDS-PAGE using pre-cast gels (4–20%, Bio-Rad) in TGS buffer (Bio-Rad, Sweden). The proteins were transferred to nitrocellulose membranes (Bio-Rad, Sweden) using the TransBlot Turbo system from Bio-Rad. The membranes were subsequently blocked for 1 h with skim milk at 3% (w/v) in PBS, then washed 3 × 10 min in PBS supplemented with 0.1% (w/v) Tween 20 (PBS-T). Then, blots were incubated with primary antibodies in PBS-T, as mentioned above, overnight. Following this, we incubated the blots with secondary antibodies for 2 h. After the secondary antibody, we washed three times with PBS-T, and then the blots were developed using ECL Clarity (Bio-Rad) according to the manufacturer’s protocol and imaged using the ChemiBlot XRS+ system from Bio-Rad.

### ELISA plates

MesoScale (MSD) plates were used to evaluate the cytokine levels (proinflammatory panels for IFN-γ, IL-1β, IL-2, IL-4, IL-5, IL-6, IL-8, IL-10, IL-12 and TNF-α) in culture media and in blood- and brain-soluble fractions from both human and mouse samples. To measure the cytokines in mouse and human soluble fractions, we pulled together 50 µg of the S1 and S2 fraction. MSD plates were also used to measure the levels of Aβ38/40/42, phosphorylated tau and total tau in the soluble and insoluble fraction from the brain extract from WT, Gal3KO, 5xFAD and 5xFAD/Gal3KO mice. Serial dilutions of the soluble and insoluble fractions were tested to obtain an accurate measure of the protein levels. 1 μg of protein from the soluble fraction was diluted to evaluate Aβ38/40/42 levels, and 0.3 µg of protein from the insoluble fraction was diluted to evaluate Aβ38/40/42 levels. The plates were developed using the 4 × reading buffer diluted to a factor of 1 × with distilled water, and the plates were read using the QuickPlex Q120 reader from Mesoscale. The detection ranges of the different cytokines measured were as follows: IL1β (1670–0.408 pg/mL), IL4 (1660–0.405 pg/mL), IL12 (32,200–7.86 pg/mL), IL10 (3410–0.833 pg/mL), IFN-γ (938–0.229 pg/mL), IL2 (2630–0.642 pg/mL), IL5 (967–0.236 pg/mL), IL6 (5720–1.40 pg/mL), KC/GRO (1980–0.483 pg/mL) and TNF-α (627–0.153 pg/mL). The Aβ40 detection range was 15,100–3.69 pg/mL, and the Aβ42 detection range was 2280–0.557 pg/mL. ELISA plates from R&D (DY008 and DY1197-05) were used to measure the levels of gal3 (detection range 1000–15.6 pg/mL) in culture media. The protocol was carried out according to the manufacturer’s protocol. FluoStar Optima reader (BMG Labtech) was used to read the gal3 assay.

### Immunohistochemistry and immunofluorescence

#### Immunohistochemistry

Mice were transcardially perfused under deep anesthesia with 4% paraformaldehyde and PBS, pH 7.4. The brains were removed, cryoprotected in sucrose and frozen in isopentane at − 15 °C. They were then cut in 40-μm thick slices in the coronal plane on a freezing microtome. The slices were serially collected in wells containing cold PBS and 0.02% sodium azide. Free-floating sections from 5xFAD and 5xFAD/Gal3KO mice were first treated with 3% H_2_O_2_/10% methanol in PBS, pH 7.4 for 20 min to inhibit endogenous peroxidases and then with an avidin–biotin blocking kit (Vector Labs, Burlingame, CA, USA) for 30 min to block endogenous avidin, biotin and biotin-binding proteins. For single immunolabeling, sections were immunoreacted with one of the following primary antibodies: anti-Aβ42 rabbit polyclonal (1:5000 dilution, Abcam), anti-amyloid precursor protein (APP) rabbit polyclonal (1:20,000 dilution, Sigma), anti-CD45 rat monoclonal (clone IBL-3/16, 1:500 dilution, AbD Serotec), anti-galectin3 (gal3) goat polyclonal (1:3000 dilution, R&D) over 24 or 48 h at room temperature. The tissue-bound primary antibody was detected by incubating for 1 h with the corresponding biotinylated secondary antibody (1:500 dilution, Vector Laboratories) and then followed by incubating for 90 min with streptavidin-conjugated horseradish peroxidase (Sigma-Aldrich) (dilution 1:2000). The peroxidase reaction was visualized with 0.05% 3-3-diaminobenzidine tetrahydrochloride (DAB, Sigma-Aldrich), 0.03% nickel ammonium sulfate and 0.01% hydrogen peroxide in PBS. After using DAB, some sections immunolabeled for APP, CD45 or gal3 were incubated for 3 min in a solution of 0.2% of Congo red. Sections were mounted on gelatine-coated slides, air-dried, dehydrated in graded ethanol, cleared in xylene and covered with DPX (BDH) mounting medium, omitting the primary antisera controlled for specificity of the immune reactions.

#### Immunofluorescence

For immunofluorescence, free-floating sections were first incubated with primary antibody followed by the corresponding Alexa secondary antibody (1:500 dilution, Invitrogen). Sections were embedded in autofluorescence eliminator reagent (Merck Millipore), following the manufacturer’s recommendations, to eliminate fluorescence emitted by intracellular lipofuscin accumulation. Tissue was incubated with the specific primary antibody for 24 h, and, the following day, the brain sections were rinsed for 1 h in PBS containing 0.1% Triton X-100. After incubating for 1 h with the corresponding secondary antibody (1:500, Alexa antibodies, Invitrogen), the sections were rinsed again with PBS containing 0.1% Triton X-100 for 60 min. Then, brain sections were mounted in glycerol 50% for visualization. For staining with thioflavin-S (Sigma-Aldrich), sections were first washed in PBS containing 0.1% Triton X-100 and then incubated for 5 min with 0.5% thioflavin-S. Next, we washed the sections for 5x10 min in PBS containing 0.1% Triton X-100. The brain sections were mounted in glycerol 50% for visualization. The fixed tissue was examined in a confocal laser microscope (Leica SP5 II), under a Olympus BX-61 epifluorescent microscope and in an inverted ZEISS LSM 7 DUO confocal laser-scanning microscope using a 20x air objective with a numerical aperture of 0.5. All images were obtained under similar conditions (laser intensities and photomultiplier voltages) and usually on the same day. Morphometric analysis of the fluorescently labeled structures was performed offline with Fiji ImageJ software (W. Rasband, National Institutes of Health). Areas for the specific antibodies were determined automatically by defining outline masks based on brightness thresholds from maximal projected confocal images [3]. Finally, the phagosome area and the circularity of plaques (suppl. Fig. 6c, d, online resource 6) were measured as described previously [65].

### Plaque loading quantification

Plaque loading was defined as the percentage of hippocampal CA1 region stained for Aβ (with anti-Aβ42). Quantification of extracellular Aβ content in neurites was done as previously described [51, 55]. Images were acquired with a Nikon DS-5 M high-resolution digital camera connected to a Nikon Eclipse 80i microscope. The camera settings were adjusted at the start of the experiment and maintained for uniformity. Digital 4 × (plaques) images from 6- and 18-month-old 5xFAD and 5xFAD/Gal3KO mice (4 sections/mouse; *n* = 6–7/age/genotype) were analyzed using Visilog 6.3 analysis program (Noesis, France). The hippocampal area (CA1 or thalamus) in each image was manually outlined, leaving out the pyramidal and granular layers in the case of APP quantification. Then, plaque areas within the hippocampal regions were identified by level threshold that was maintained throughout the experiment for uniformity. The color images were converted to binary images with plaques. The loading (%) for each transgenic mouse was estimated and defined as (sum plaque area measured/sum hippocampal area analyzed) × 100. The sums were taken over all slides sampled, and a single burden was computed for each mouse. The mean and standard deviation (SD) of the loadings were determined using all the available data. Quantitative comparisons were carried out on sections processed at the same time with the same batches of solutions.

### Hippocampal Aβ injections in wild-type mice

Aβ monomers (10 μM) and Aβ monomers together with gal3 (10 μM) were pre-incubated for 1 h at 37 °C prior to the intracerebral injections. A volume of 2 μl was injected (0.5 μl/min) in the dentate gyrus. In the left hemisphere, we injected only Aβ monomers, and, in the right hemisphere, we injected Aβ monomers with gal3. Two months after injections, the mice were sacrificed and transcardially perfused with 4% PFA. The brains were postfixed in 4% PFA overnight followed by sucrose saturation (25% in PBS). Brains were sectioned by a microtome (30 µm) and stained for ThioS, 6E10, GFAP, NeuN and Iba1.

### Stochastic optical reconstruction microscopy (STORM)

The samples were the same that we used for the confocal images (see the protocol in immunofluorescence staining section in the “Materials and methods” section), except that we changed the buffer and the detection device. Images were acquired as previously described by Van der Zwaag et al. [56]. Briefly, images were acquired using a Nikon N-STORM system configured for total internal reflection fluorescence (TIRF) imaging. STORM buffer contains 10 mM Tris pH 8, 50 mM NaCl an oxygen scavenging system (0.5 mg/mL glucose oxidase, Sigma-Aldrich), 34 μg/mL catalase (Sigma), 5% (w/v) glucose and 100 mM cysteamine (Sigma-Aldrich). Excitation inclination was tuned to adjust the focus and to maximize the signal-to-noise ratio. Fluorophores were excited by illuminating the sample with the 647 nm (~ 125 mW) and 488 nm (~ 50 mW) laser lines built into the microscope. Fluorescence was detected by means of a Nikon APO TIRF 100 ×/1.49 Oil W.D. 0.12 mm. Images were recorded onto a 256 × 256 pixel region of a EMCCD camera (Andor Ixon3 897). Single-molecule localization movies were analyzed with NIS element Nikon software.

### Immunofluorescence of human sections

Endogenous peroxidases were deactivated by incubating the samples in a peroxidase block for 15 min with gentle agitation. The sections were then washed (3 × 15 min) in 0.1 M KPBS, after which they were incubated in blocking buffer (5% goat serum blocking with 0.1 M KPBS and 0.025% Triton-X) for at least 1 h with gentle agitation. The sections were then washed (3 × 15 min) in 0.1 M KPBS, and the primary antibody was added (1:300). The sections were then incubated at 4 °C overnight with gentle agitation. The sections were washed (3 × 15 min) in 0.1 M KPBS, after which poly-HRP secondary antibody was added. The sections were then incubated for 1 h at room temperature. For triple Iba1/gal3/Aβ immunofluorescence, sections were first incubated with the primary antibodies followed by the corresponding Alexa 647/488/555 secondary antibodies (1:1000 dilution, AlexaFluor, Life Technologies). Sections were embedded in 0.6 g Sudan Black (Sigma) dissolved in 70% ethanol. The sections, after mounting and drying on slides, were incubated in the sudden black solution for 5 min. Subsequently, the sections were washed in PBS and mounted with mounting medium. The camera settings were adjusted at the start of the experiment and maintained for uniformity. A Nikon Eclipse Ti confocal microscope (Nikon, Japan) and NIS elements software (Nikon, Japan) were used to take 20x magnification pictures and for the final collage.

### Fluorescent anisotropy

#### Production of recombinant human galectins

Recombinant human galectins (i.e., gal3 wild-type and gal3 R186S mutant) were produced in *E. coli* BL21 Star (DE3) cells and purified by affinity chromatography on lactosyl-sepharose columns, which has been previously described by Salomonsson et al. [49].

#### Establishment of the affinity between galectins and TREM2

A fluorescence anisotropy (FA) assay was used to determine the affinity of recombinant TREM2 for wild-type or mutant gal3 in solution. The method has previously been described in detail by Sörme et al. for saccharides and synthetic small-molecule galectin inhibitors [53]. In short, increasing concentrations of galectins are first tittered against a fixed concentration of saccharide probe (0.02 µM). When this is done, the anisotropy value increases from a value when probe is free in solution (*A*_0_) to a value when all probe molecules are bound to galectins (*A*_max_). To establish the dissociation constant (*K*_d_) values between TREM2 and gal3 wild-type or R186S mutant, a competitive variant of the FA assay was used. In this assay, increasing concentrations of TREM2 were tittered against fixed concentrations of galectin and probe (see below for details). By obtaining the anisotropy values for the different TREM2 concentrations, together with the values for *A*_max_ and *A*_0_, the *K*_d_ values could be calculated according to the equations presented in Sörme et al. [53].

The FA of the fluorescein-conjugated probes was measured using a PheraStarFS plate reader and PHERAstar Mars version 2.10 R3 software (BMG, Offenburg, Germany). The excitation wavelength used was 485 nm, and the emission was read at 520 nm. All experiments were performed in PBS at room temperature (~ 20 °C). The anisotropy values for each data point were read in duplicate wells of 386-well plates (at a total volume of 20 µl). *K*_d_ values were calculated as weighted mean values from concentrations of TREM2 that generated between 20 and 80% inhibition, where inhibition values of approximately 50% had the highest impact on the mean value.

*Gal3 (wild type) affinities* Experiments were performed with gal3 at a concentration of 0.30 µM and the fluorescent probe 3,3′-dideoxy-3-[4-(fluorescein-5-yl-carbonylaminomethyl)-1*H*-1,2,3-triazol-1-yl]-3′-(3,5-dimethoxybenzamido)-1,1′-sulfanediyl-di-β-d-galactopyranoside at 0.02 µM [50].

*Gal3 (R186S mutant) affinities* Experiments were performed with gal3 R186S at a concentration of 2 µM and the fluorescent probe 2-(fluorescein-5/6-yl-carbonyl)aminoethyl-2-acetamido-2-deoxy-α-d-galactopyranosyl-(1–3)-[α-l-fucopyranosyl-(1–2)]-β-d-galactopyranosyl-(1–4)-β-d-glucopyranoside at 0.02 µM [13].

### TREM2 and galectin-3 3D modeling

The extracellular domain of TREM2 (white) with tetratennary N-glycan (stick-model) and gal3 CRD (yellow) was modeled. Mutations in TREM2 leading to an increased risk of AD are blue, and those leading to Nasu–Hakola disease (NHD) are red. The C-terminus of the extracellular fragment that normally links further to the transmembrane domain is green (suppl. Fig. 7c).

The TREM2 model is from pdb 5 ELI, which was published by Ref. [29].

A tetranatennary N-glycan was modeled in at the single N-glycosylation site using the GlyCam server, Woods Group (2005–2018) GLYCAM Web. Complex Carbohydrate Research Center, University of Georgia, Athens, GA, USA (http://glycam.org). The model of bound gal3 CRD was from a structure in complex with LacNAc (pdb IKJL, [52], which was superimposed on the terminal LacNAc of the N-glycan. The picture was made with The PyMOL Molecular Graphics System, Version 2.0 Schrödinger, LLC.

### Gene array

Hippocampal samples from 5xFAD, 5xFAD/Gal3KO, WT and Gal3KO mice at 6 and 18 months were collected and snap-frozen in dry ice to carry out the mRNA evaluation. mRNA was extracted using the RNAeasy Mini Kit (Qiagen) according to the manufacturer’s protocol. The extraction was performed automatically using the QIAcube device from Qiagen. RNA concentration was subsequently quantified using a NanoDrop 2000C. Samples with a RIN value under 8.7 were not included. cDNA synthesis was performed using Superscript Vilo cDNA Synthesis (ThermoScientific) according to the manufacturer’s protocol. TaqMan^®^ OpenArray^®^ Mouse Inflammation, TaqMan^®^ OpenArray^®^ Real-Time PCR Master and TaqMan^®^ OpenArray^®^ Real-Time PCR were used to perform the qPCR. Real-Time PCR Open Array from Applied Biosystems was used to read the Open Array 384-well plates, which were used to perform the qPCR.

### Gene array analysis

Differently expressed genes were identified using the data from the HTqPCR assay assessed in the *Openarray* platform (Qiagen). Statistical analysis was performed using the software *DataAssist v3.01.* The maximum CT permitted was 35. First, we sorted the data based on the gene fold change and then we converted the data to Log2FC. We compared 5xFAD mice at 6 and 18 months to WT mice and also compared the 5xFAD mice with the 5xFAD Gal3KO mice at 6 and 18 months. We selected genes with a Log2FC value between ± 2 at 6 months and ± 4 at 18 months. Out of 629 genes, 95 were selected at 6 months and 106 genes at 18 months. For the analysis of the main pathways affected by the lack of gal3 at 6 and 18 months in our 5xFAD mouse model, we used network analysis along with KEGG and the Reactome database.

### Behavioral tests

The Morris water maze test tests spatial acquisition memory and was conducted in a pool consisting of a circular tank (180 cm diameter) filled with opaque water at 20 °C ± 1 °C. A platform (15 cm diameter) was submerged 10 mm under the water surface. A white curtain with specific distal visual cues surrounded the water maze. White noise was produced from a radio centrally positioned above the pool to avoid the use of auditory cues for navigation. Spatial learning sessions were conducted over ten consecutive days with four trials per day. Each trial was started by introducing the mouse, facing the pool wall, at one of four starting points in a quasi-random fashion to prevent strategy learning. Each mouse remained on the platform for 30 s before transfer to a heated waiting cage. During all acquisition trials, the platform remained in the same position. On the day following the last learning trial, a 60 s probe test was conducted, during which the platform was removed from the pool. All mouse movements were recorded using a computerized tracking system that calculated distances moved and latencies required for reaching the platform (ANY-maze 5.2).

### Total RNA extraction and qPCR

Total RNA and proteins were extracted using TriPure Isolation Reagent (Roche). RNA integrity (RIN) was determined using RNA Nano 6000 (Agilent). The RIN was 8.5 ± 0.5. RNA was quantified using a NanoDrop 2000 spectrophotometer (ThermoFischer, Spain).

### Retrotranscription and quantitative real-time RT-PCR

Retrotranscription (RT) (4 μg of total RNA) was performed with the High-Capacity cDNA Archive Kit (Applied Biosystems). For real-time qPCR, 40 ng of cDNA was mixed with 2 × Taqman Universal Master Mix (Applied Biosystems) and 20x Taqman Gene Expression assay probes (Applied Biosystems, supplemental). Quantitative PCR reactions (qPCR) were done using an ABI Prism 7900HT (Applied Biosystems). The cDNA levels were determined using GAPDH as the housekeeping gene. Results were expressed using the comparative double-delta *C*_t_ method ($2^{{ - \Delta \Delta C_{\text{t}} }}$). Δ*C*_t_ values represent GAPDH normalized expression levels. ΔΔ*C*_t_ was calculated using 6-month-old WT mice samples.

#### Taqman probes

Iba1 (Ref. Mm00479862_g1), CD45 (Ref. Mm01293577_m1), CD68 (Ref. Mm03047343_m1), TREM2 (Ref. Mm04209424_g1), Cx3Cr1 (Ref. Mm02620111_s1), GAPDH (Ref. Mm99999915_g1), IL-6 (Ref. Mm00446190_m1), TNFa (Ref. Mm00443258_m1), GFAP (Ref. Mm01253033_m1).

### Genetic association analysis

#### Datasets

Genotypic datasets from four genome-wide association studies (GWAS) were used in this study: (a) the Murcia study [2]; (b) the Alzheimer’s Disease Neuroimaging Initiative (ADNI) study [41]; (c) the GenADA study [36]; and (d) the NIA study [60] (for GWAS dataset details, see supplementary information). The Murcia study was previously performed by researchers from our group. Datasets from the ADNI, GenADA, and NIA studies were obtained from dbGAP (http://www.ncbi.nlm.nih.gov/gap), Coriell Biorepositories (http://www.coriell.org/) or ADNI (http://adni.loni.ucla.edu/). Prior to the genetic association analysis, each dataset (Murcia, ADNI, GenADA, NIA and TGEN) was subjected to both an extensive quality control analysis and a principal component analysis. In addition, since different platforms were used in the five GWAS analyzed, we imputed genotypes using HapMap phase 2 CEU founders (*n* = 60) as the reference panel. This approach has been previously described [2, 8, 37]. Overall, a total of 2252 cases and 2538 controls were included in the meta-analysis.

#### SNP selection

To select single-nucleotide polymorphisms (SNPs) within the *LGALS3* gene, including 1000 pb upstream and downstream of that genetic region, we used the UCSC Table Browser data retrieval tool [26], release genome assembly: Mar. 2006 (NCBI36/hg18), from the UCSC Genome Browser database (http://genome.ucsc.edu/) [21]. Selected SNPs were extracted from GWAS datasets using Plink v1.06 software [47].

#### Linkage disequilibrium blocks

Linkage disequilibrium (LD) blocks were determined along the genomic regions studied using Haploview software [5] and genotyping data from the largest dataset used (NIA dataset).

#### Association analyses

Unadjusted single-locus allelic (1 *df*) association analysis within each independent GWAS dataset was carried out using Plink software. We combined data from these four GWAS datasets using the meta-analysis tool in Plink selecting only those markers common to, at least, three studies. For all single-locus meta-analyses, fixed effect models were employed when no evidence of heterogeneity was found, otherwise random effect models were employed.

All selected SNPs were located close to 3′end of the *LGALS3* gene. Because all SNPs belonged to the same linkage disequilibrium block, multiple test correction was not applied. Thus, the *p* value threshold was established as 0.05.

### Supplementary information regarding the GWAS datasets used

The Murcia study was designed as a new case–control GWAS of the Spanish population. In this study, 1128 individuals were genotyped using an Affymetrix NspI 250K chip. A sample of 327 sporadic Alzheimer’s disease (AD) patients diagnosed as possible or probable AD in accordance with NINCDS–ADRDA criteria by neurologists at the Virgen de Arrixaca University Hospital in Murcia (Spain) and 801 controls with unknown cognitive status from the Spanish general population were included. The Alzheimer’s Disease Neuroimaging Initiative (ADNI) longitudinal study was launched in 2003 by the National Institute on Aging (NIA), the National Institute of Biomedical Imaging and Bioengineering (NIBIB), the Food and Drug Administration (FDA), private pharmaceutical companies and non-profit organizations as a $60 million, 5-year public–private partnership. The primary goal of ADNI has been to test whether serial magnetic resonance imaging, positron emission tomography, other biological markers and clinical and neuropsychological assessment can be combined to measure the progression of mild cognitive impairment (MCI) and early AD. Determination of sensitive and specific markers of very early AD progression is intended to aid researchers and clinicians in developing new treatments and monitoring their effectiveness, as well as to lessen the time and cost of clinical trials. The principal investigator of this initiative is Michael W. Weiner, MD, VA Medical Center and University of California, San Francisco. ADNI is the result of efforts of many co-investigators from a broad range of academic institutions and private corporations, and subjects have been recruited from over 50 sites across the US and Canada. The initial goal of ADNI was to recruit 800 adults, aged 55–90, to participate in the research, approximately 200 cognitively normal older individuals to be followed for 3 years, 400 people with MCI to be followed for 3 years and 200 people with early AD to be followed for 2 years. For up-to-date information, see http://www.adni-info.org. The GenADA study included 801 cases that met the NINCDS–ADRDA and DSM-IV criteria for probable AD and 776 control subjects without a family history of dementia that were genotyped using the Affymetrix 500K GeneChip Array set. The NIA Genetic Consortium for Late Onset Alzheimer’s Disease (LOAD) study originally included 1985 cases and 2059 controls genotyped with the Illumina Human 610Quad platform. Using family trees provided in the study, we excluded all related controls and kept one case per family, resulting in a total of 1077 cases and 876 controls.

Some data used in preparation of this article were obtained from the Alzheimer’s disease neuroimaging initiative (ADNI) database (http://adni.loni.ucla.edu/). As such, the investigators within the ADNI contributed to the design and implementation of ADNI and/or provided data but did not participate in analysis or writing of this report. A complete listing of ADNI investigators can be found at https://adni.loni.usc.edu/wp-content/uploads/how_to_apply/ADNI_Acknowledgement_List.pdf.

### Human material

All the human material used was obtained from the Lund University Hospital, Neuropathology Unit (suppl. Table 1, online resource 8; Elisabet Englund, elisabet.englund@med.lu.se) and The Netherlands Institute for Neuroscience, Amsterdam, The Netherlands (Inge Huitinga, i.huitinga@nin.knaw.nl; suppl. Table 2, online resource 9). Written informed consent for the use of brain tissue and clinical data for research purposes was obtained from all patients or their next of kin in accordance with the International Declaration of Helsinki. Medisch Ethische Toetsingscommissie (METc) of VU University has approved the procedures for brain tissue collection, and the regional ethical review board in Lund has approved the study. All human data were analyzed anonymously.

### Antibodies

Antibodies used for this study: anti-rabbit iNOS primary antibody (1:5000, Santa Cruz), anti-rat gal3 antibody (1:3000, M38 clone from Hakon Leffler’s lab, in-house antibody), anti-goat gal3 antibody (1:1000, R&D Systems), anti-mouse actin antibody 1:10,000 (Sigma-Aldrich), anti-human Aβ antibody (1:5000, Covance), anti-rabbit Iba-1 antibody (1:500, Wako), anti-mouse TLR4 antibody (1:1000, Santa Cruz), anti-mouse NLRP3 antibody (1:5000, Adipogen), anti-rabbit C83 antibody (369) (1:1000, Gunnar Gouras Laboratory, BMC, Lund, Sweden), TREM2 antibody 1:500 (AF1729, anti-sheep) anti-rabbit IDE-1 antibody (1:1000, Calbiochem), anti-Rabbit p-Tau (pTau181, 1:1000, Santa Cruz), anti-mouse Aβ (1:1000, Sigma-Aldrich), anti-CD45 rat monoclonal (clone IBL-3/16, 1:500, AbD Serotec), anti-CD68 (1:500, eBiosciences) and anti-Clec7a rabbit monoclonal (1:500, abcam). Secondary antibodies used for western blot were anti-rabbit, anti-mouse, anti-goat and anti-rat from Vector Labs. Secondary antibodies used for immunofluorescence were raised in donkey and were anti-rabbit, anti-goat, anti-mouse and anti-rat from Life Technology (AlexaFluor).

### Inhibitor used in our study

The inhibitor used for the experiments in suppl. Fig. 5 (online resource 5) was 1,1′-sulfanediyl-bis-3-deoxy-3-4-3-fluorophenyl-1*H*-1,2,3-triazol-1-yl-β-d-galactopyranoside [45] (inhibitor 1). It was synthesized and characterized as reported previously [16]. The purity was determined to be 97.3%, according to UPLC-analysis (Waters Acquity UPLC system, column Waters Acquity CSH C18, 0.5 ml/min H_2_O-MeCN gradient 5-95% 10 min with 0.1% formic acid).

## Results

### Galectin-3 is expressed in microglial cells positioned next to amyloid plaques in AD human cortical samples and 5xFAD mice

**Fig. 1:**
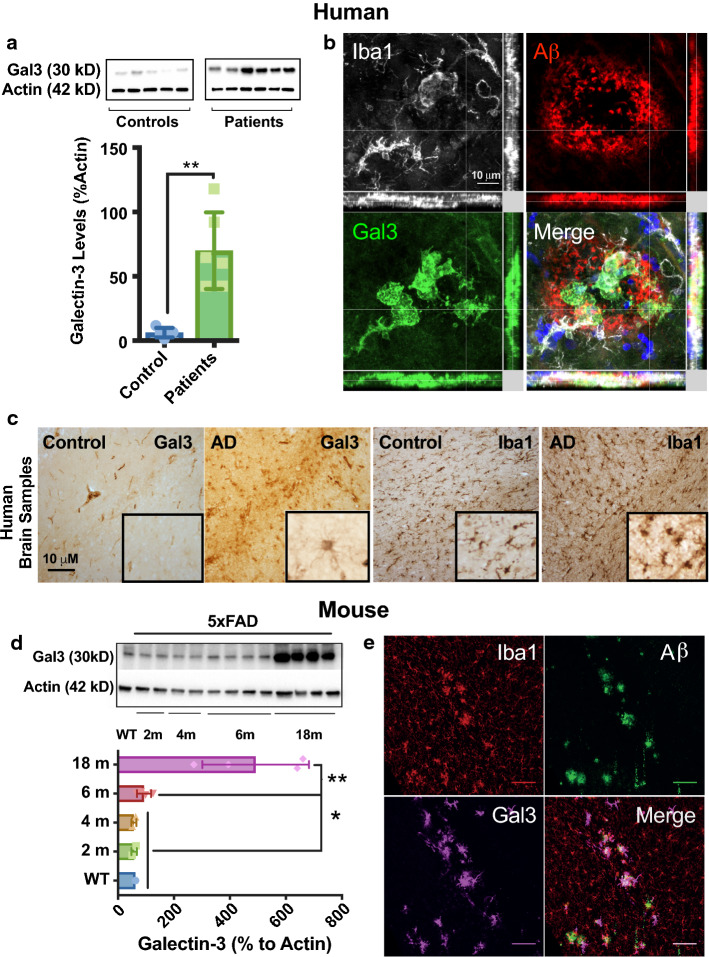
Galectin-3 is increased in human and mouse AD brains and marks a microglial phenotype associated with Aβ plaques. **a** Western blot analyses of cortex from human AD cases (*n* = 6) and age-matched healthy controls (*n* = 5). **b** Galectin-3 (gal3) staining mainly coincided with Iba1^+^ microglia found around Aβ plaques in human AD brains. **c** Immunohistochemistry showed high levels of gal3 in microglia in AD brains, as compared to Iba1-staining (low levels of gal3 was detected in association to blood vessels). **d** Gal3 protein was significantly upregulated in the cortex of 5xFAD mice in a time-dependent fashion (WT, 6 months old). **e** Gal3 expression was found in Iba1^+^ cells around Αβ plaques. Statistical significance was calculated by one-way ANOVA with Tukey’s correction (**d**) or Student’s *t* test (**a**). *p < 0.05; **p < 0.01. Data are shown as mean ± SD. The human AD cases are described in suppl. Tables S1 and S2 (online resources 8 and 9)

We first evaluated the levels of gal3 in cortical sections from AD patients and age-matched healthy controls (Fig. 1a). We found a ten-fold increase of gal3 in AD patients compared to controls. Next, we stained for gal3, Aβ and microglia (Iba1) in AD and healthy cortical sections (Fig. 1b, c). The expression of gal3 was mostly absent in the control samples with a faint staining in brain blood vessels (Fig. 1c). However, in the AD cortical samples, there was robust gal3 staining in Iba1^+^ cells (Fig. 1c), which was strictly confined to Aβ plaque-associated microglia (Fig. 1b). To further confirm our observations, we used 5xFAD mice, a transgenic AD mouse model, and found the expression of gal3 significantly upregulated in a time-dependent fashion from 6 to 18 months (Fig. 1d; suppl. Fig. 1, online resource 1). In 5xFAD brains, gal3 was typically found in microglial cells associated with Aβ plaques (Fig. 1e). No noticeable gal3-expressing microglia was found neither in hippocampus nor in cortex in 18-month-old WT mice (data not shown).

We also analyzed two early stages of 5xFAD mice, including 6 weeks (no sign of plaque is detected at this age) and 10 weeks (first early plaques appear in the subiculum) (suppl. Fig. 1b, c, online resource 1). This temporal analysis demonstrated the appearance of gal3-expressing microglia at very early stages of the disease, before pre-plaque formation. At 6 weeks, it was evident that the gal3-expressing microglial cells were located close to APP expressing neurons even with no sign of Aβ plaque deposition (suppl. Fig. 1, online resource 1). Despite this close association, further experiments are required to decipher its biological significance. We also investigated if other cell types in 5xFAD mice expressed gal3 by looking at GFAP-labeled astrocytes and NeuN-labeled neurons. This study demonstrated that plaque-associated microglia is the predominant cell type expressing gal3. Yet, 13% of reactive astrocytes expressed gal3 in 6-month-old 5xFAD mice. That figure dropped to less than 2% in 18-month-old 5xFAD mice (suppl. Fig. 2, online resource 2). On the other hand, neurons expressing gal3 were rarely observed even after analyzing several hippocampal sections. The expression of neuronal gal3 was, therefore, considered very exceptional (suppl. Fig. 2c, online resource 2).

### Single-nucleotide polymorphisms (SNP) associated with the gal3 gene increase the risk of developing AD

We performed genetic association studies using a SNP meta-analysis approach of the *LGALS3* gene, which encodes gal3. A total of 60 SNPs was identified in the *LGALS3* genetic region. Only five SNPs were genotyped in at least three GWAS (suppl. Fig. 3a, suppl. Table 3, online resource 3 and 10) and were found to be in high linkage disequilibrium (*d*′ > 0.96; suppl. Fig. 3b, online resource 3). Interestingly, all five SNPs were associated with an increased AD frequency (*p* < 0.03; suppl. Table 3, online resource 10), suggesting a potential causal role of gal3 in the development of AD.

### Galectin-3 regulates amyloid-dependent microglial activation

**Fig. 2:**
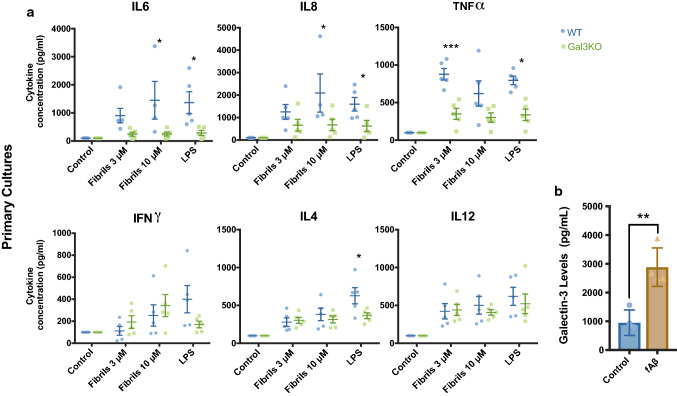
Galectin-3 deficiency/inhibition reduces the microglial inflammatory response in vitro. **a** Reduced cytokine levels in culture medium from Gal3KO primary microglial cultures compared to WT after fΑβ treatment for 12 h. **b** WT primary microglial cultures increase the release of gal3 upon stimulation with fAβ. In vitro experiments represent a minimum of three independent experiments. Statistical significance was calculated by Student’s t-test (**b**), or one-way ANOVA with Tukey’s correction (**a**) *p < 0.05; **p < 0.01; ***p < 0.001. Data are shown as mean ± SD

To investigate the role of gal3 in microglial activation in AD, we first evaluated the inflammatory response in BV2 microglial cells. fAβ was characterized prior to the experiments (suppl. Fig. 5a, online resource 5). Our preparation was tested for LPS endotoxin as well as for cell toxicity (suppl. Fig. 5b, c, online resource 5). BV2 cells challenged with fAβ-activated microglial cells, inducing the expression of iNOS and NLRP3 (suppl. Fig. 5d, online resource 5) and the production and release of proinflammatory cytokines (suppl. Fig. 5e, online resource 5). Next, we challenged BV2 cells with 10 μM fAβ along with increasing concentrations of gal3 inhibitor (10 and 25 μM) for 12 h. The conditioned media was collected, and inflammatory-related cytokines were measured. Notably, the release of proinflammatory cytokines TNFα, IL12 and IL8 was significantly reduced in response to gal3 inhibition in a concentration-dependent manner (suppl. Fig. 5f, online resource 5). The same was true for iNOS, a classical proinflammatory marker as measured by western blot (suppl. Fig. 5g, online resource 5). We also evaluated the levels of insulin degrading enzyme 1 (IDE-1) in BV2 cells. IDE-1 is an enzyme involved in Aβ degradation [48]. IDE-1 was downregulated in BV2 cells challenged with fAβ in a concentration-dependent fashion (suppl. Fig. 5i, left, online resource 5). Strikingly, with gal3 inhibition, the downregulation of Aβ-induced IDE-1 turned into a significant upregulation in a manner dependent on inhibitor concentration (suppl. Fig. 5i, online resource 5). To confirm our in vitro data, we evaluated the inflammatory response using primary microglial cultures from WT and Gal3KO mice. Primary microglial cultures were challenged with fAβ at 3 and 10 μM. Similar to BV2 cells, the lack of gal3 reduced the release of proinflammatory cytokines, such as IL6, IL8 and TNFα (Fig. 2a). Other cytokines, such as IFNγ, IL4 and IL12, were not affected by the lack of gal3. We next analyzed the effect of gal3 on the phagocytosis of either Aβ monomers (mAβ) or Aβ fibrils (fAβ) by primary microglia. To achieve this, primary microglial cells were either pretreated (30 min) or simultaneously with gal3 (1 µM) upon challenge with mAβ or fAβ. Microglial uptake of Aβ was increased when mAβ and gal3 were added simultaneously (suppl. Fig. 2e, online resource 2). Interestingly, when using pretreatment of fAβ (suppl. Fig. 2f, online resource 2), we found a reduced microglial uptake, suggesting a perplex role of gal3 in Aβ phagocytosis. We have previously demonstrated the ability of gal3 to be released by proinflammatory microglia in response to TLR activation, which was shown to be a determinant feature in driving microglia-related immune responses [9, 64]. Given the strong response to either gal3 deletion or gal3 inhibition in the prevention of the fAβ-induced microglia proinflammatory activation, we next analyzed if cultured microglia challenged with fAβ actively release gal3. Gal3 levels were analyzed in conditioned media using ELISA. This analysis demonstrated a prominent release of gal3 in response to fAβ in both primary microglia cultures and BV2 cells (Fig. 2b and suppl. Fig. 5h, online resource 5).

### Galectin-3 regulates microglial activation in 5xFAD mice

**Fig. 3:**
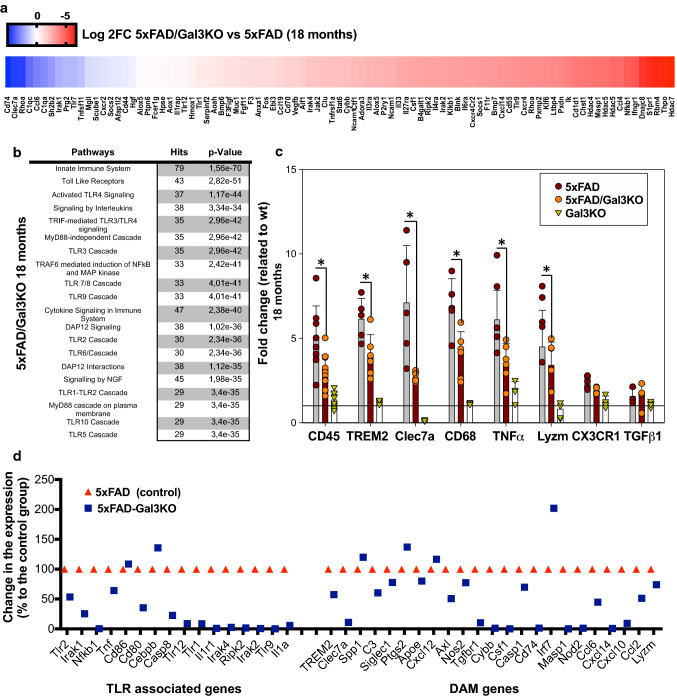
Galectin-3 reduces the microglial inflammatory response in vivo mainly through TLR and DAP12 pathways. **a** Heat map of the 100 most upregulated inflammatory genes in 18-month-old 5xFAD mice vs. WT and how these genes are altered in 5xFAD/Gal3KO mice. Data expressed in Log2FC. **b** Inflammatory pathways affected in hippocampi of 5xFAD/Gal3KO mice compared to 5xFAD mice. **c** Expression of homeostatic and proinflammatory microglial genes in 5xFAD and 5xFAD/Gal3KO using qPCR in aged mice (18 months). **d** Main TLR-associated genes and disease-associated microglia (DAM) genes affected in aged mice (18 months); red triangle is 5xFAD reference gene expression and blue square is 5xFAD/Gal3KO gene expression. Data are expressed in folds ΔCT. Statistical significance was calculated by one-way ANOVA with Tukey’s correction. *p < 0.05. Data are shown as mean ± SD

To examine the role of gal3 in the neuroinflammatory response, WT, 5xFAD mice and crossbred 5xFAD/Gal3KO mice were compared. Transcriptional profiles of hippocampal tissue were obtained by running high-throughput qPCR against a 632-gene mouse inflammation panel. WT and Gal3KO mice showed quite similar profiles, suggesting a limited role of gal3 in promoting inflammation in the absence of pathology. In 5xFAD mice, compared to WT, a significant number of genes were affected, especially in 18-month-old mice, in which 125 out of 629 immune genes were highly upregulated, including: complement factors, chemokines, interleukin receptors and toll-like receptors (suppl. Fig. 4, online resource 4). Overall, the whole immune response was highly attenuated in 5xFAD/Gal3KO mice (suppl. Fig. 4, online resource 4). Interestingly, genes associated with the recently characterized neurodegenerative disease-associated phenotype (DAM microglia [27, 30]), such as Clec7a, Csf1, Cd74, Cxcl10 and Cybb (Fig. 3a, b, d), were downregulated in 5xFAD/Gal3KO mice compared to 5xFAD mice. qPCR analysis of homeostatic (Cx3cr1, TGFβ1) and DAM/reactive microglia (CD45, TREM2, Clec7a, CD68, TNFα and Lysozyme M) confirmed the instrumental role of gal3 in driving the brain immune response in 5xFAD mice (Fig. 3). To identify potential cell signaling pathways related to gal3 in 5xFAD mice, we used NetworkAnalyst (http://www.networkanalyst.ca) [61]. Most affected pathways were related to TLR receptors and DAP12 signaling (Fig. 3b, d). DAP12 has been described as a TREM2 adaptor and plays a critical role in the switch from homeostatic to disease-associated microglian [27, 30]. Our data anticipate a key role of gal3 in driving microglial activation in AD through regulation of different microglial pattern-recognition receptors, including TLRs and TREM2.

### The lack of galectin-3 reduces AD pathology in 5xFAD mice

**Fig. 4:**
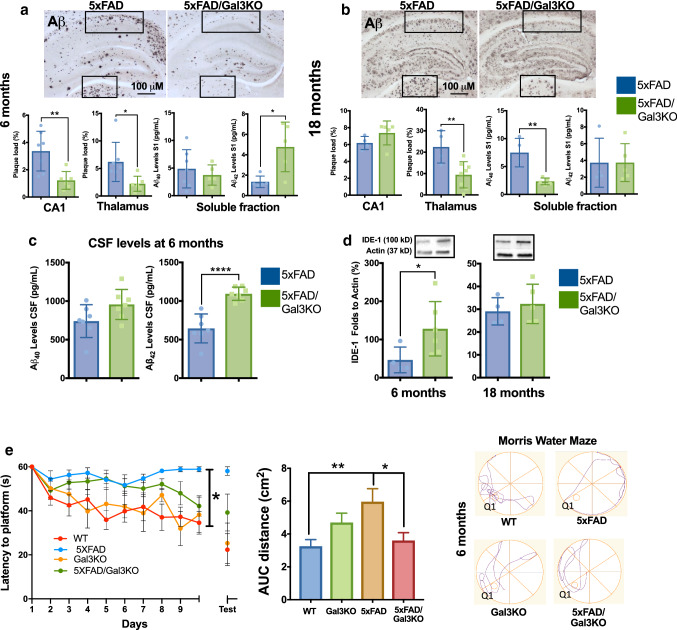
Lack of galectin-3 reduces AD pathology in 5xFAD mice. Aβ load in hippocampus (CA1) and thalamus at 6 months (**a**) and 18 months (**b**) with Aβ load analyzed in 5xFAD and 5xFAD/Gal3KO mice (% in frames). Soluble fractions of Αβ40 and Αβ42 levels in cortical S1 fraction were measured (pg/ml). **c** Αβ40 and Αβ42 levels in CSF samples at 6 months (pg/mL**)**. **d** IDE-1 levels in 5xFAD and 5xFAD/Gal3KO mice at 6 and 18 months. **e** Spatial memory analyzed by Morris water maze test. Center, distance traveled (meters) as an integrated distance (*AUC* area under the curve). Left, latency to the platform during training trials. Right, representative runs of probe trial day. Q1, quadrant where the platform was located on training trial. Statistical significance was calculated by two-way (with Bonferroni’s post hoc) (**e**) and Student’s t-test (**a**–**e**). *p < 0.05; **p < 0.01; ***p < 0.001; ****p < 0.0001. Data are shown as mean ± SEM

Once we confirmed the important role of gal3 in driving AD-associated microglial immune responses, we next analyzed the effect of gal3 in AD pathogenesis. There was clear age-dependent Aβ deposition in 5xFAD mice (Fig. 4). Aβ deposition in aged animals (18 months) was higher; an indication that Aβ clearance becomes saturated in aged 5xFAD mice. Consequently, to detect a potential effect of gal3 in Aβ burden, we analyzed the effect of gal3 gene deletion in two well-defined areas, the hippocampal CA1 area (high rate of Aβ deposition) and the thalamus (low rate of Aβ deposition), in 5xFAD mice at 6 and 18 months of age. Notably, the plaque burden was significantly reduced in 5xFAD mice lacking gal3 at 6 months in the CA1 region (Fig. 4a). At 18 months, we could not find any difference between the groups, possibly due to oversaturated plaque deposits in the hippocampi (Fig. 4b). In agreement with this view, a significant reduction in Aβ plaque deposition was found in the thalamus in 5xFAD/Gal3KO mice at 6 and 18 months compared to aged-matched 5xFAD controls (Fig. 4a, b). We also performed an analysis of amyloid plaque morphology (size, circularity and perimeter). We compared thioflavin-S-positive plaques in 5xFAD/Gal3KO and 5xFAD mice at 6 and 18 months. This analysis first demonstrated that aged animals exhibited a near threefold increase in plaque area in both experimental groups when compared to 6-month-old animals. Notably, at 18 months, we found a significant reduction in plaque perimeter and circularity in 5xFAD/Gal3KO mice compared to 5xFAD mice (suppl. Fig. 6c, online resource 6). Next, we measured cortical Aβ42 and Aβ40 levels in soluble brain fractions. The levels of Aβ40 were significantly lower in 5xFAD/Gal3KO (vs. 5xFAD) brains at 18 months but not at 6 months (Fig. 4a, b). Conversely, Aβ42 levels were significantly higher in 5xFAD/Gal3KO (vs. 5xFAD) brains at 6 months (Fig. 4a) but with no change at 18 months (Fig. 4b). In the insoluble Aβ fraction, we found reduced Aβ40 levels in 5xFAD/Gal3KO mice at 6 months (suppl. Fig. 6a, online resource 6). Changes in Aβ40 and Aβ42 levels were not due to a change of APP expression as 5xFAD and 5xFAD/Gal3KO mice showed comparable levels at both ages measured (suppl. Fig. 6b, online resource 6). Aβ is cleared from the brain by different mechanisms, including microglia phagocytosis, drainage to the ventricular system and activity of extracellular proteases, such as IDE-1 [18]. We found a significant increase of Aβ42 CSF levels in 5xFAD/Gal3KO mice compared to 5xFAD mice (Fig. 4c), mimicking the clinical situation in which Aβ in the CSF is inversely related to the Aβ plaque load in AD patients. Interestingly, hippocampal IDE-1 levels were increased in 5xFAD/Gal3KO mice at 6 months compared to 5xFAD mice (Fig. 4d), confirming our in vitro data (suppl. Fig. 5i, online resource 5). Since phagocytosis is also involved in Aβ clearance, we analyzed CD68-labeled phagosome areas that have been shown to correlate with in vivo Aβ phagocytosis [65] at 6 months and 18 months in 5xFAD and 5xFAD/Gal3KO mice. No significant differences were found between both experimental groups at any of the ages examined (suppl. Fig. 6d, online resource 6).

Taken together, our data are suggestive of an important negative role of gal3 in Aβ clearance in 5xFAD mice. This view is sustained by the higher CSF levels of Aβ42 and increased expression of extracellular IDE-1 in 5xFAD/Gal3 KO mice when compared to 5xFAD mice (Fig. 4).

Having established that gal3 is a main driver of the AD-associated inflammatory response and that gal3 deficiency reduces Aβ plaque burden, we next studied the impact of gal3 deficiency on cognitive behavior. The cognitive ability was evaluated using the Morris water maze memory test. Remarkably, the lack of gal3 in 5xFAD/Gal3KO mice resulted in better cognitive performance compared to 5xFAD mice as the 5xFAD/Gal3KO mice were able to navigate and reach the platform using a shorter path (Fig. 4e).

### Galectin-3 drives microglia reactivity in Aβ plaques

**Fig. 5:**
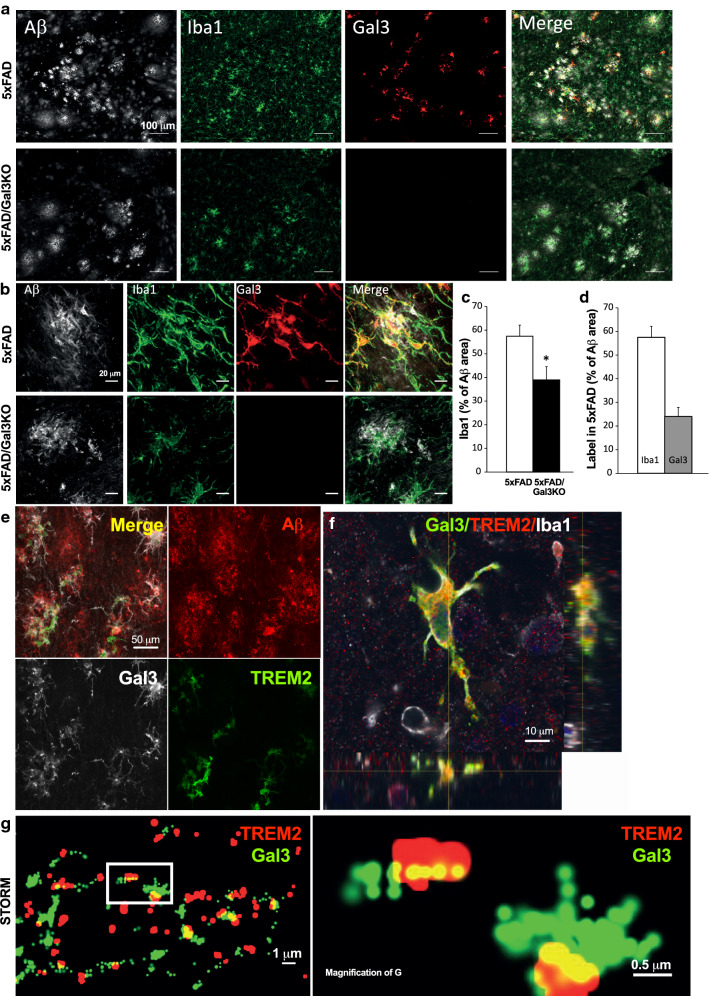
Galectin-3 colocalizes with TREM2. **a**, **b** Iba1^+^ cells expressing galectin-3 (gal3) around Aβ plaque in 5xFAD mice. **c** Reduced number of Iba1^+^ microglial cells around Αβ plaques in 5xFAD/Gal3KO mice compared to 5xFAD mice (% of Αβ area). **d** Number of Iba1^+^ cells expressing gal3 in 5xFAD (% of Αβ area). **e**, **f** Gal3 and TREM2 in plaque-associated microglia in the brain of 5xFAD mice reveals colocalization of gal3 and TREM2. **g** Gal3 and TREM2 colocalization in 5xFAD mouse brain using STORM microscopy. Statistical significance was calculated by Student’s *t* test. *p < 0.05. Data are shown as mean ± SEM. All images were taken in 5xFAD mice at 18 months

In light of our findings that gal3-reactive microglia are strictly confined to Aβ plaques and that gal3 deficiency impairs the inflammatory response and Aβ plaque load, we next analyzed microglial coverage on the surface of amyloid plaques in cortex from both 5xFAD and 5xFAD/Gal3KO mice at 18 months of age. First, we found that 5xFAD/Gal3KO mice showed reduced Iba1 immunoreactivity in the cortex when compared to 5xFAD mice (Fig. 5a). These differences were particularly evident in plaque-associated microglia in 5xFAD/Gal3KO mice (Fig. 5b). Remarkably, gal3 deficiency significantly reduced Iba1-labeled microglial clusters around Aβ plaques (Fig. 5c). Still, an analysis of microglial coverage over plaque surfaces showed that a high subset of plaque-associated microglia (~ 50%) expressed high levels of gal3 (Fig. 5d). Interestingly, these findings are, to some extent, similar to the findings seen in studies of 5xFAD/TREM2KO mice [39, 59, 65], yet, substantial differences are evident between 5xFAD lacking either gal3 or TREM2. Thus, contrary to that seen in 5xFAD lacking gal3, 5xFAD/TREM2KO mice exhibits increased Aβ deposition in the hippocampal formation [59]. Further, microglia lacking TREM2 fail to colocalize in Aβ plaques [59], an ability present in microglia lacking gal3. Taken together, our data suggest that gal3-associated biological effects may be associated with TREM2 signaling without excluding other significant biological pathways (i.e., TLR signaling).

### Galectin-3 acts as a TREM2 ligand

Recent single-cell transcriptomic analyses of microglia under different conditions of neurodegeneration, including AD, have revealed that gal3 is one of the most upregulated genes in processes that have been suggested to be TREM2-dependent [30, 38]. We have demonstrated that gal3 is released by reactive microglial cells [9] and may thus interact with different receptors controlling key functions of microglial cells, including TLR4 [38] and MerTK [43]. One of the main pathways altered in 5xFAD/Gal3KO mice was related to DAP12 signaling (Fig. 3b). Since DAP12 is a downstream regulator of TREM2, we wondered if gal3 could act as an endogenous TREM2 ligand. To test our hypothesis, we first performed TREM2, gal3 and 6E10 staining on 5xFAD brain sections and found that the majority of TREM2-labeled microglia were gal3^+^ (83.4 ± 5.7% and 94.0 ± 3.02% at 6 and 18 months, respectively), an indication that gal3 immunoreactivity may label the DAM phenotype [27, 30] (Fig. 5e, suppl. Fig. 7a, online resource 7). To further support this view, we performed Clec7a immunostaining on 5xFAD brain sections. Clec7a has been identified as a specific microglial marker associated with both the DAM phenotype [27] and the microglial neurodegenerative phenotype [30]. Our confocal analysis demonstrated that plaque-associated microglia express the markers Clec7a and gal3 (suppl. Fig. 2d, online resource 2), thus supporting the view that DAM express gal3. Our analysis also demonstrated a striking cellular colocalization of TREM2 and gal3 in microglial cells around Aβ plaques (Fig. 5e, f, suppl. Fig. 7a, online resource 7). Despite the clear relation between TREM2 and gal3 in microglial cells-associated amyloid plaques, further experiments (such as single-cell analysis) are required to fully elucidate how gal3 is related to DAM microglia.

We next used quantitative high-resolution confocal microscopy and super-resolution stochastic optical reconstruction microscopy (STORM) to determine whether TREM2 and gal3 physically interact in brain sections from 5xFAD mice. Our ultrastructural analysis clearly revealed the physical interaction between gal3 (green) and TREM2 (red) over the membrane surface of microglia (see yellow dots in Fig. 5g). Further, we incubated brain sections from 5xFAD/Gal3KO mice with recombinant gal3 to further analyze gal3 binding in situ. This experiment demonstrated the binding of gal3 to TREM2^+^ but not to TREM2^−^/Iba1^+^ microglial cells (suppl. Fig. 7b, online resource 7).

**Fig. 6:**
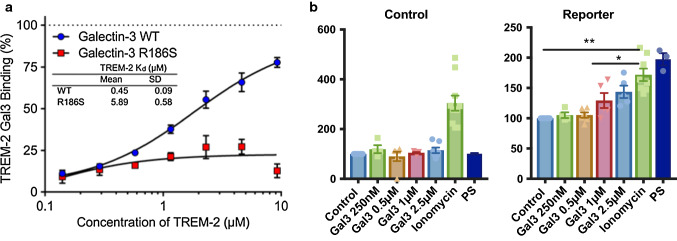
Galectin-3 interacts with TREM2 through its carbohydrate-binding domain. **a** Fluorescent anisotropy assay for galectin-3 (gal3)/TREM2 interaction. Data are presented as % of TREM2–gal3 binding (gal3, WT and mutant gal3 with deficient carbohydrate-binding domain, R186S) and fluorescent probe interaction, by increasing concentrations of TREM2, together with the calculated *K*_d_ values for the gal3/TREM2 interaction (*n* = 2). **b** Control and DAP12 reporter cell lines were stimulated with increasing concentrations of gal3 (250 nM–2.5 µM), ionomycin and phosphatidylserine (PS). Statistical significance was calculated by Student’s *t* test or one-way ANOVA with Bonferroni’s post hoc test. *p < 0.05; **p < 0.01. Data are shown as mean ± SD

We further assessed the direct TREM2–gal3 interaction by determining the ability of soluble TREM2 to inhibit the binding of a fluorescent glycosylated probe to gal3 as measured by fluorescence anisotropy (Fig. 6a). This analysis demonstrated a strong interaction between WT gal3 and TREM2 with a *K*_d_ value of 450 ± 90 nM (Fig. 6a), which was in the same range as other preferred glycoproteins [11, 12]. A mutant gal3 (R186S, deficient in the carbohydrate-binding domain with severely reduced affinity for LacNAc-structures) [49], in comparison, weakly bound to TREM2 (*K*_d_ of 5900 ± 600 nM; Fig. 6a). This means that gal3 interacts strongly with TREM2, with a *K*_d_ in the same range as, for example, some serum glycoproteins such as transferrin and haptoglobin [11, 12]. The data also suggest that the interaction between gal3 and TREM2 is carbohydrate dependent. The hypothesized interaction was modeled based on previously reported TREM2 and gal3 structures (suppl. Fig. 7c, online resource 7). Next, we tested whether gal3 could affect TREM2 signaling using a TREM2–DAP12 reporter cell line, and we found that added gal3 triggered TREM2–DAP12-dependent signaling in a dose-dependent manner (Fig. 6b). Overall, our data demonstrate that gal3 is an endogenous ligand for TREM2.

### Galectin-3 induces the formation of insoluble Aβ aggregates following injections of Aβ in the hippocampi of WT mice

**Fig. 7:**
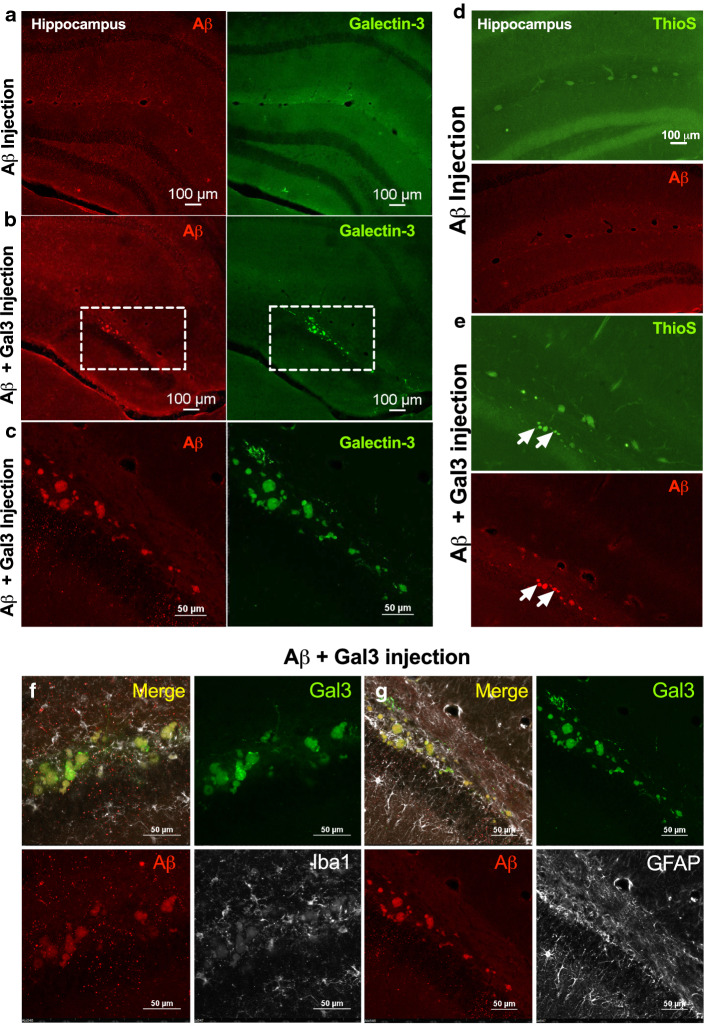
Galectin-3 induces the formation of insoluble Αβ aggregates following injections of Αβ monomers in the hippocampi of WT mice. Αβ monomers were injected with galectin-3 (Gal3) (Aβ + Gal3) or without (Aβ) after 1 h Aβ monomers incubation w/o gal3 into the right or left hippocampi of WT mice, respectively. **a** Staining for Αβ and gal3 in the left hippocampus (only Aβ monomers injected). **b**, **c** Staining for Αβ and gal3 in the right hippocampus (Aβ + gal3 injected). Dashed frames in **b** are magnified and shown in **c**. **d** Thioflavin-S and Αβ staining of the left hippocampus (only Aβ injected). **e** Thioflavin-S and Αβ staining of the right hippocampus (Aβ + gal3 injected). White arrows point to gal3 and thioflavin-S^+^ aggregates. **f**, **g** Iba1 and GFAP immunoreactivity in the right hippocampus (Αβ + gal3 were injected)

We have previously demonstrated that gal3 can be released by reactive microglial cells in vitro and in vivo [9]. In this study we provide convincing evidence that primary microglia and BV2 cells actively release gal3 when challenged with fAβ and that gal3 may act as an endogenous TREM2 agonist. Intriguingly, TREM2 has been associated with Aβ plaque compaction [65], and, hence, the possibility emerged that gal3 may have a role in Aβ aggregation. To address this, Aβ42 monomers (10 µM) were incubated alone or in the presence of gal3 (10 µM) for 1 h at 37 °C (suppl. Fig. 6f, online resource 6). A ThT assay was performed under the same conditions and demonstrated that fAβ, which reached the maximal ThT fluorescence intensity, was the predominant form of Aβ aggregates used for the in vivo injections (suppl. Fig. 6f, online resource 6). Two microliter of the prepared Aβ was injected in either the left (Aβ + gal3) or the right hippocampi (Aβ alone) of WT mice. The analysis was performed 2 months post-injection. The injection of Aβ alone failed to induce Aβ aggregation in the injected hippocampi (Fig. 7a). In sharp contrast and remarkably, Aβ-like plaques were evident in the hippocampi injected with Aβ + gal3 (Fig. 7b, c). These Aβ aggregates were positive for gal3, Aβ (detected with 6E10 antibody) and thioflavin-S (Fig. 7d, e), which demonstrate the amyloid structure of the Aβ deposits. These amyloid deposits were surrounded by Iba1^+^-labeled microglia (Fig. 7f) and GFAP^+^ reactive astrocytes (Fig. 7g). Animals injected with Aβ alone did not present aggregate deposits or microglial/astrocyte activation (suppl. Fig. 6e, online resource 6). Our data shed light on the role of gal3 as microglial-associated agent involved in Aβ aggregation.

## Discussion

Recent findings suggest an important role of neuroinflammation in neurodegenerative diseases [18]. Thus, selective mutations in microglia/myeloid-specific genes, including the gene encoding TREM2, have been associated with AD [16]. Experimental AD studies have suggested that TREM2 is instrumental in neuroinflammation [24, 35] and drives DAM microglia [27, 30]. Gal3, a carbohydrate-binding protein, is one of the most upregulated genes associated with DAM [30, 34, 38]. Holtman et al. (2015) anticipated gal3 as a gene strongly related to microglial DAM phenotype [22]. To study the role of gal3 in AD pathogenesis, we have analyzed human brains from AD patients and 5xFAD mice lacking gal3. The main conclusions from our study are (1) gal3 protein is increased tenfold in human AD brains and is mostly restricted to plaque-associated microglia in humans and 5xFAD mice, (2) certain SNPs linked to the *LGALS3* gene are associated with AD, (3) gal3 deficiency reduces Aβ plaque burden and the overall proinflammatory response and plaque size and improves cognitive performance in 5xFAD/Gal3KO mice, (4) gal3 is an endogenous TREM2 ligand, (5) gal3 is released in response to fAβ and (6) gal3 interacts with Aβ, affecting amyloid plaque morphology. Hence, gal3 emerges as a central upstream regulator of AD-associated pathology.

Given the relationship between innate immune system-related genes and AD incidence [18, 19], deciphering how Aβ aggregates trigger neuroinflammation is of critical importance. For this purpose, we first analyzed the role of gal3 in the Aβ-induced inflammatory response in microglial cells. To this end, we took advantage of both chemical inhibition and gene deletion. Gal3 inhibition robustly reduced fAβ-induced iNOS expression in BV2 cells, an effect that was extended to proinflammatory cytokines, including TNFα, IL6, IL8 and IL12, which we confirmed in primary microglia cultures from WT and Gal3KO mice. These data suggest that gal3 is a critical alarmin that amplifies the Aβ-induced proinflammatory response. Since inefficient clearance of Aβ may play a determinant role in AD pathogenesis [67], we analyzed the effect of gal3 in two major mechanisms involved in Aβ clearance: Aβ phagocytosis and IDE-1 levels, a key metalloprotease involved in Aβ degradation by microglia [48]. Gal3 was able to alter Aβ phagocytosis in vitro showing a complex role of gal3 in Aβ phagocytosis depending on Aβ species (monomers or fibrils) or if pretreated or not (see suppl. Fig. 2e–f, online resource 2). Hence, further experiments are needed to clarify the exact role of gal3 in the whole dynamic process of Aβ phagocytosis and clearance. We found gal3 deficiency to increase IDE-1 levels in vitro and in vivo, suggesting a detrimental role of gal3 in Aβ clearance. Supporting this, CSF levels of Aβ42 were significantly higher in 5xFAD/Gal3KO as compared with 5xFAD. Overall, our study demonstrates an instrumental role of gal3 in driving the fAβ-induced proinflammatory response and in contributing to a deleterious effect in Aβ clearance.

Recent transcriptomic analysis of microglia at the single-cell level has identified a common disease-associated microglia phenotype, which has been suggested to be driven by TREM2 and apoE [27, 30, 38]. Interestingly, Krasemann et al. (2017) identified gal3 as one of the most upregulated genes in plaque-associated microglia, supporting our findings in human AD brains and 5xFAD mice [30]. Recently, Yang et al. overexpressed TREM2 in 5xFAD mice (5xFAD/TREM2) and found gal3 as one of the main genes affected by TREM2 overexpression [34], suggesting that gal3 plays an important role in microglial function under disease conditions. We generated 5xFAD/Gal3KO mice to answer whether gal3 plays a role in microglia-associated AD pathogenesis and to test if gal3 signaling is associated with TREM2. To answer this question, we performed an inflammatory gene array, demonstrating an age-dependent inflammatory response, involving complement components, chemokines, interleukin receptors, toll-like receptors and DAM genes in 5xFAD mice. This inflammatory response was highly attenuated in 5xFAD/Gal3KO mice, thus confirming gal3 as a master regulator of AD-associated brain immune responses. Pathway analysis in mice at 6 and 18 months of age identified TLR and DAP12, a TREM2 signaling adaptor protein, as one of the most significant pathways associated with gal3 in the 5xFAD mice. This gene expression data suggest an important role of gal3 in the AD neuroinflammatory response, perhaps by stimulating NFκb-related regulators whereby TLR4, and/or other TLRs [9, 25] or glycoproteins, such as TREM2 [14, 65], are involved. Despite the clear connection between gal3-, TLR- and TREM2-related pathways, the regulation of these pathways is not known, and further experimentation is needed.

Consequently, we investigated whether gal3 interacts with TREM2, a key receptor that has been suggested as a driver of the DAM phenotype. Our confocal microscopy study demonstrated a remarkable cellular colocalization of TREM2 and gal3 in microglial cells around Aβ plaques and a near 100% correspondence between both microglial markers, an indication that gal3 specifically labels DAM. This view was further supported by Clec7a immunostaining, a recognized DAM marker. STORM microscopy demonstrated that TREM2 and gal3 physically interact on microglial processes, most likely at the cell membranes of DAM microglia. We also incubated sections from 5xFAD/Gal3KO mice with recombinant gal3 to analyze the potential binding between TREM2 and extracellular gal3. This assay demonstrated the ability of gal3 to selectively bind to TREM2^+^ microglia but not to homeostatic microglia. Direct interaction between gal3’s carbohydrate recognition domain and TREM2 was demonstrated by fluorescent anisotropy with a *K*_d_ value of 450 ± 90 nM, which was in the same range as other preferred glycoproteins [11, 12]. The ability of gal3 to stimulate TREM2 was finally confirmed by a TREM2–DAP12 reporter cell line. Overall, our data suggest that the switch from homeostatic microglia to DAM is accompanied by significant upregulation of both TREM2 and gal3. Gal3 may thus bind to and activate different microglial receptors including TREM2 (present study), TLR4 [9], IGFR [33] and MerTK [43]. In fact, because gal3 is relatively promiscuous in its interactions with glycoproteins, it may be behind the chronic and detrimental activation of microglia in AD. Regardless of this possibility, what our study demonstrates is that the pleiotropic activity of gal3 drives important amyloid-associated immune responses.

Additionally, our study has uncovered an unexpected role of gal3 as a powerful Aβ binding agent. We have previously demonstrated the ability of LPS-induced reactive microglia to release gal3 [9, 64]. More recently, we have demonstrated a significant increase of gal3 levels in CSF from mice exposed to traumatic brain injury [64], an indication that gal3 is released by reactive microglia. In this study, we provide evidence that gal3 is actively released by microglia in response to a fAβ challenge. In APP mice, gal3 is present in the extracellular space and associated with amyloid fibrils (unpublished). Taken together, the possibility that gal3 directly interacts with Aβ to affect aggregation becomes plausible. To test this possibility, we incubated Aβ monomers with or without gal3 for 1 h at 37 °C and injected 2 µl of each condition into either the left or right hippocampi of WT mice. A ThT assay demonstrated fAβ as the predominant form of Aβ present in both injections. Aβ deposition was analyzed 2 months after injections. While no Aβ deposition was found in animals injected with Aβ monomers alone, co-injection of Aβ monomers and gal3 resulted in evident insoluble Aβ aggregates, suggesting that gal3 is directly involved in Aβ plaque formation. To our knowledge, this is the first report showing insoluble Aβ aggregates long after Aβ brain injections in WT mice. Thus, Meyer-Luehmann et al. [40] injected brain homogenates from aged APP mice in the hippocampus of WT mice and found no evidence of Aβ aggregates 4 months after injection. In contrast, injections of the same brain extracts in AD transgenic mice led to robust deposition of Aβ [15, 28, 40, 44]. Further, repetitive Aβ injections into the hippocampus of WT mice demonstrated that Aβ deposits are drastically reduced from day 1 to day 7 after injections [15].

Recently, Heneka et al. have demonstrated that ASC specks released from reactive microglia physically interact with Aβ, acting as an Aβ cross-seeding agent [57]. Eliminating microglia has been shown to prevent plaque formation in APP transgenic mice [54], suggesting that factors released from microglia may seed amyloid plaques. Our study reinforces the view that reactive microglia play a critical role in Aβ plaque dynamics and associated immune responses by releasing Aβ cross-seeding agents (i.e., ASC specks) and gal3. Aβ plaque formation is believed to precede the appearance of clinical symptoms, so elucidating the earlier molecular mechanism involved in Aβ plaque formation appears critical for the establishment of early promising therapeutic strategies aiming at halting the development of AD early. Gal3-inhibition appears to be a promising Aβ therapeutic target. The ability of gal3 to drive proinflammatory fAβ-associated immune responses and hinder Aβ clearance makes this protein a strategic upstream regulator of AD pathology. Indeed, the pathogenic role of gal3 was confirmed in adult 5xFAD mice lacking gal3 as those mice had a significantly lower Aβ load and ameliorated cognitive/spatial memory deficits, which was observed in the Morris water maze test.

Our results suggest that *LGALS3* gene variants affect the risk of developing AD, as indicated by the 5 SNPs in the *LGALS3* gene that we found to be related to increased AD frequency. However, according to the GWAS catalogue (https://www.ebi.ac.uk/gwas/), none of the variants reported in suppl. Table 3 (online resource 10) or those in linkage disequilibrium with them (suppl. Fig. 3b, online resource 3), have been previously associated with AD. This could be due to the fact that the GWAS approach requires large samples to detect modest effects, partly due to the multiple testing corrections applied. However, the impact of these *LGALS3* SNPs appears to be similar to other SNPs included in the GWAS, supporting a role for this gene in the development of AD. Our meta-analysis only comprised those SNPs belonging to a linkage disequilibrium block located at the 5′-end of the *LGALS3* gene, therefore, we do not know if other regions have genetic variants that could lead to stronger effects. At present it is difficult to speculate in what way the SNPs of gal3 is altering the function or expression of the protein. Further replication studies covering the entire genetic region of this locus will be necessary to confirm our results.

## Conclusions

In conclusion, we provide evidence that gal3 is a central upstream regulator of the microglial immune response in AD. It drives proinflammatory activation of microglia in response to fAβ along with impairment of fAβ degradation and clearance. Our study has additionally uncovered another exciting feature of gal3 disease biology that appears important in AD pathogenesis. Gal3 plays a role as an endogenous TREM2 ligand, a key receptor driving microglial activation in AD. As a result, gal3 inhibition may be a potential pharmacological approach to counteract AD.

## Electronic supplementary material

Below is the link to the electronic supplementary material.

Suppl. Figure 1 Galectin-3 protein expression in 5xFAD mice. a Immunohistochemistry reveals galectin-3 (gal3) upregulation (in red) in coronal brain sections from 5xFAD mice at 6 and 18 months of age (right) compared to 9-month-old WT mice (left). Gal3^+^ cells were absent in WT mice. b-c Gal3^+^ microglial cells are present at very early time points, 6 weeks (before plaque deposition, c) and 10 weeks (first plaque deposits, b) in 5xFAD mice (PDF 10044 kb)

Suppl. Figure 2 Rare galectin-3 protein expression in non-microglial cells. a Colocalization of GFAP (red) and galectin-3 (gal3) (green) in the hippocampal molecular layer in brain sections from 5xFAD mice, suggesting that astrocytes can be positive for gal3. b GFAP and gal3^+^ cell number decrease from 6 to 18 months in 5xFAD mice. c Colocalization of NeuN (green) and gal3 (red) in the pyramidal cell layer of the hippocampus in 5xFAD mouse brain sections suggests that some neurons can be positive for gal3. d Clec7a (red) and gal3 (green) colocalize in 5xFAD mice at 18 months. White arrow points to Clec7a-Gal3 colocalization. e Primary microglia treated with w/o gal3 (1 µM) before (30 min) or simultaneous with mΑβ (200 nM). f Primary microglia treated with w/o gal3 (1 µM) before (30 min) or simultaneous with fΑβ (200 nM). mΑβ, monomeric Αβ; fΑβ fibrillar Αβ (Aβ1-42 tagged with Fluor 647). Values expressed in % mAβ or fAβ uptake (vs. control) (*n* = 4) Statistical significance was calculated by one-way ANOVA with Tukey’s post hoc test **p < 0.01. HC = Hippocampus. Data shown as in mean ± SD (PDF 3861 kb)

Suppl. Figure 3 Single nucleotide polymorphisms associated with the gene for galectin-3 (*LGALS3*). a A total of 60 SNPs (single nucleotide polymorphisms) were identified in the *LGALS3* genetic region. Only five SNPs were genotyped in at least three GWAS (suppl. Table 3, online resource 10). Five SNPs within the *LGALS3* gene (including 1,000 bp upstream and downstream of the genetic region) were studied in relation to AD frequency in five different AD cohorts (Murcia, ADNI, GenADA, NIA and TGEN), including a total of 2,252 AD cases and 2,538 controls for the meta-analysis. b All the five studied SNPs were in high linkage disequilibrium (d´ > 0.96), suggesting non-random association of the studied alleles (PDF 160 kb)

Suppl. Figure 4 Inflammatory genes exhibiting the most altered expression in 5xFAD/Gal3KO mice compared to 5xFAD mice. a Upregulated genes in the hippocampi of 5xFAD mice compared to WT (>3 folds ΔCT) at 6 months old. b Upregulated genes in the hippocampi of 5xFAD mice compared to WT (>5 folds ΔCT) at 18 months old. c Downregulated genes in 5xFAD/Gal3KO mice compared to 5xFAD mice (50% cut-off of value of repressed genes) at 6 months. d Downregulated genes in 5xFAD/Gal3KO mice compared to 5xFAD mice (50% cut-off of value of repressed genes) at 18 months. Genes in red are specifically related to TLR- and TREM2-signaling (PDF 890 kb)

Suppl. Figure 5 Characterization of the fibrils used and the activated microglia. a Electron transmission microscopy pictures of Αβ fibrils used to activate microglial cells *in vitro* following fibril generation. b Endotoxin assay used to measure the endotoxin levels in our Αβ fibril preparations. The levels measured were within the standards (ng/mL) and unlikely to affect the experiments. c Cell viability assay used to test cell viability following a challenge with the Αβ fibril preparation ± the gal-3 inhibitor used in our *in vitro* experiments. Values are expressed in absorbance 450 nm as mitochondrial activity. d iNOS levels in BV2 cells challenged with Αβ (3 and 10 μΜ) for 12 h (left). NLRP3 levels in BV2 cells challenged with Αβ (3 and 10 μΜ) for 12 h (right). LPS (1 μg/ml) was used as a control. Proteins levels are shown relative to actin levels (right). e Cytokines released into the medium by BV2 cells challenged with Αβ (3 and 10 μΜ) for 12 h. LPS (1 μg/mL) was used as a positive control. f Reduced cytokine levels culture medium in microglial BV2 cell cultures challenged with galectin-3 (gal3) inhibitor and fΑβ (fΑβ, 10 μΜ) for 12 h. g Reduced iNOS levels in BV2 cells treated with gal3 inhibitor together with fΑβ 10 μΜ for 12 h. h BV2 microglial cells increase gal3 release (culture medium) following stimulation with fΑβ. i IDE-1 levels in BV2 cells challenged with fΑβ was reduced (left, 10 μΜ), but increased when adding gal3 inhibitor (10 and 25 µM) along with fΑβ (10 μΜ) for 12h (right). *In vitro* experiments represent a minimum of 3 independent experiments. Statistical analysis was done using one-way ANOVA with Bonferroni’s post hoc test. **p < 0.01; ***p < 0.001; ****p < 0.0001. Data are shown as mean ± SD (PDF 2900 kb)

Suppl. Figure 6 Evaluation of insoluble Aβ levels, Aβ plaque morphology, phagosome formation and glial reaction after Aβ injections in WT mice. a Insoluble fraction (P3) levels. Aβ42 and Aβ40 levels were measured by ELISA. The P3 fraction was extracted from the cortex of 5xFAD and 5xFAD/Gal3KO mice at 6 and 18 months. Proteins levels are in ng/mL. b APP levels were measured by western blot at 6 and 18 months in 5xFAD and 5xFAD/Gal3KO mice. c Amyloid plaque deposit analyzed at 6 and 18 months in 5xFAD and 5xFAD/Gal3KO mice (perimeter, circularity and area of the plaque). d CD68 staining performed at 6 and 18 months in 5xFAD and 5xFAD/Gal3KO mice. Phagosome area in μm^2^ right (white arrows point to the phagosomes). e GFAP and Iba1 immunoreactivity to Αβ injected in the hippocampi of WT mice. f ThT assay performed to characterize Αβ monomers and Αβ monomers + galectin-3 preparations injected in WT mice. Statistical analysis was performed using Student’s t-test. p** < 0.01, p*** < 0.001. Data are shown as mean ± SD (PDF 10837 kb)

Suppl. Figure 7 Galectin-3 binds to TREM2 in a CRD dependent-fashion. a Galectin-3 (gal3), Aβ and TREM2 colocalized in brain slides from 18-month-old 5xFAD mice. b Exogenous galectin-3 (ExGal3) added to 5xFAD/Gal3KO brain sections preferentially labeled TREM2-enriched areas within plaque-associated microglia. The yellow arrows point to Iba1^+^ microglia not to upregulating TREM2. The blue arrows point to a TREM2-gal3 (yellow) interaction in a reactive Iba1^+^ microglial cell exhibiting morphological features of plaque-associated microglia. c Model of TREM2 (white) with tetrantennary N-glycan (stick model) and gal3 CRD (yellow). Mutations in TREM2 associated with increased risk for AD are blue, and mutations known to cause Nasu-Hakola disease (NHD) are red. The C-terminus of the fragment, which normally links further to the transmembrane domain, is green (PDF 4440 kb)

Supplementary material 1 (PDF 70 kb)

Supplementary material 1 (PDF 96 kb)

Supplementary material 1 (PDF 136 kb)
