# Correction to: Galectin-3, a novel endogenous TREM2 ligand, detrimentally regulates inflammatory response in Alzheimer’s disease

**DOI:** 10.1007/s00401-023-02549-1

**Published:** 2023-02-15

**Authors:** Antonio Boza-Serrano, Rocío Ruiz, Raquel Sanchez-Varo, Juan García-Revilla, Yiyi Yang, Itzia Jimenez-Ferrer, Agnes Paulus, Malin Wennström, Anna Vilalta, David Allendorf, Jose Carlos Davila, John Stegmayr, Sebastian Jiménez, Maria A. Roca-Ceballos, Victoria Navarro-Garrido, Maria Swanberg, Christine L. Hsieh, Luis M. Real, Elisabet Englund, Sara Linse, Hakon Leffler, Ulf J. Nilsson, Guy C. Brown, Antonia Gutierrez, Javier Vitorica, Jose Luis Venero, Tomas Deierborg

**Affiliations:** 1 Department of Experimental Medical Science, Experimental Neuroinflammation Laboratory, Lund University, 221 84 Lund, Sweden; 2 Departamento de Bioquímica y Biología Molecular, Instituto de Biomedicina de Sevilla (IBiS, HUVR/CSIC/Universidad de Sevilla), Universidad de Sevilla, Seville, Spain; 3 Departamento de Biología CelularGenética y Fisiología, Instituto de Investigación Biomédica de Málaga (IBIMA), Facultad de Ciencias, Universidad de Málaga, Málaga, Spain; 4 Department of Biochemistry, University of Cambridge, Cambridge, UK; 5 Clinical Memory Research Unit, Department of Clinical Sciences Malmö, Lund University, Malmö, Sweden; 6 Department of Biochemistry and Structural Biology, Lund University, Lund, Sweden; 7 Unidad Clínica de Enfermedades Infecciosas y Microbiología, Hospital Universitario de Valme, Seville, Spain; 8 Division of Oncology and Pathology, Department of Clinical Sciences, Lund University, Lund, Sweden; 9 Department of Laboratory Medicine, Division of Microbiology, Immunology and Glycobiology (MIG), Lund University, Lund, Sweden; 10 Department of Chemistry, Centre for Analysis and Synthesis, Lund University, Lund, Sweden; 11 Translational Neurogenetics Unit, Department of Experimental Medical Science, Wallenberg Neuroscience Center, Lund University, 221 84 Lund, Sweden; 12 Immunology Section, Department of Medicine, San Francisco VA Medical Center, UCSF School of Medicine, 4150 Clement St. 111R, San Francisco, CA 94121 USA; 13 Centro de Investigación Biomédica en Red sobre Enfermedades Neurodegenerativas (CIBERNED), Madrid, Spain

**Correction to: Acta Neuropathologica (2019) 138:251–273**

 10.1007/s00401-019-02013-z

In this article author would like to make few changes in the article text. The corrections are given below.

1. In Materials and methods section for Immunofluorescence of human sections:

In the sentence:

“The sections were then washed (3 × 15 min) in 0.1 M KPBS, after which they were incubated in blocking buffer (5% **goat** serum blocking with 0.1 M KPBS and 0.025% Triton-X) for at least 1 h with gentle agitation.”

Action: Change the word Goat for Donkey.

2. In Material and methods section for ELISA plates:

In the paragraph:

“The detection ranges of the different cytokines measured were as follows: IL1β (1670–0.408 pg/mL), IL4 (1660–0.405 pg/mL), IL12 (32,200–7.86 pg/mL), IL10 (3410–0.833 pg/mL), IFN-γ (938–0.229 pg/mL), IL2 (2630–0.642 pg/mL), IL5 (967–0.236 pg/mL), IL6 (5720–1.40 pg/mL), **KC/GRO** (1980–0.483 pg/mL) and TNF-α (627–0.153 pg/mL). The Aβ40 detection range was 15,100–3.69 pg/mL, and the Aβ42 detection range was 2280–0.557 pg/mL. ELISA plates from R&D (DY008 and DY1197-05) were used to measure the levels of gal3 (detection range 1000–15.6 pg/mL) in culture media.”

Action: Write “IL8 regarded as human functional homolog to rodent KC” between brackets next to KC/GRO.

