# Supplementary figures and images for: Galectin-3, a novel endogenous TREM2 ligand, detrimentally regulates inflammatory response in Alzheimer’s disease
Source: Acta Neuropathol. 2019 Apr 20;138(2):251–73. doi: 10.1007/s00401-019-02013-z (PMC6660511; doi:10.1007/s00401-019-02013-z)

# Suppl. Fig. 1

## Gal3 protein expression

**a**

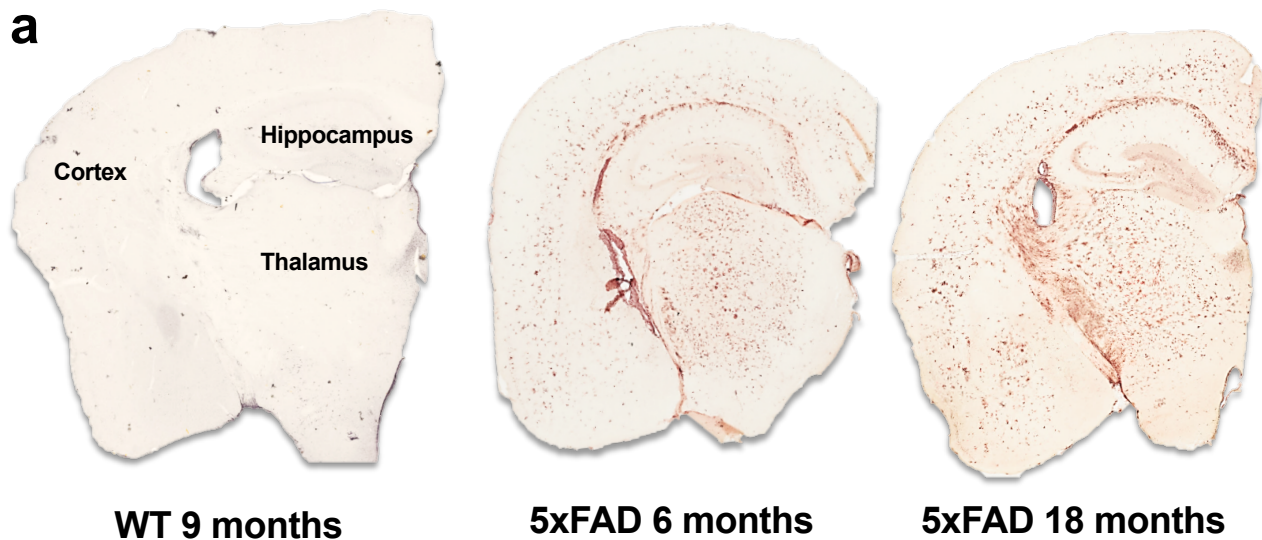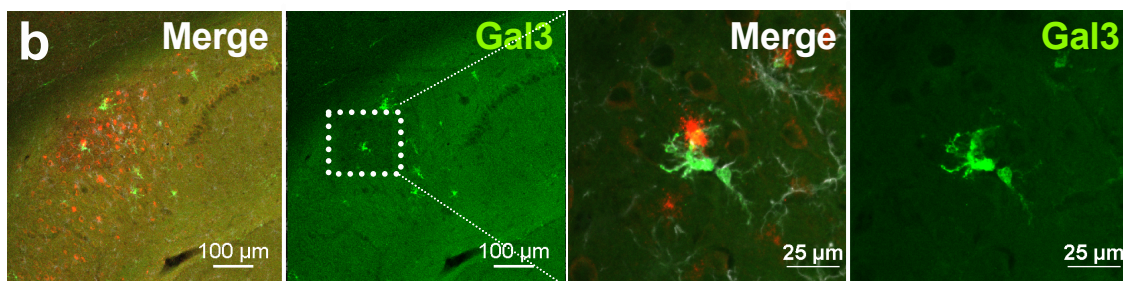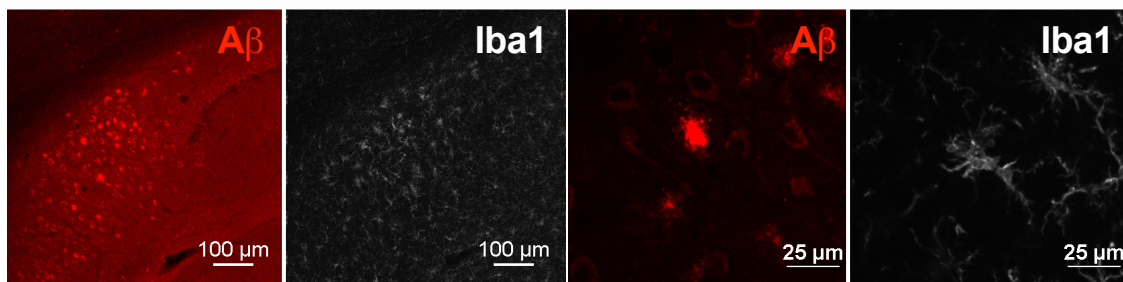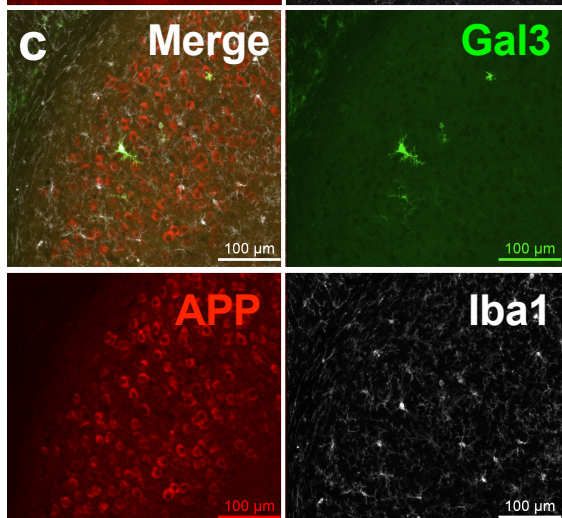

Supplement: Supplementary file 1 — Suppl. Figure 1 Galectin-3 protein expression in 5xFAD mice. a Immunohistochemistry reveals galectin-3 (gal3) upregulation (in red) in coronal brain sections from 5xFAD mice at 6 and 18 months of age (right) compared to 9-month-old WT mice (left). Gal3+ cells were absent in WT mice. b-c Gal3+ microglial cells are present at very early time points, 6 weeks (before plaque deposition, c) and 10 weeks (first plaque deposits, b) in 5xFAD mice (PDF 10044 kb) [file 401_2019_2013_MOESM1_ESM.pdf]

Suppl. Fig. 2

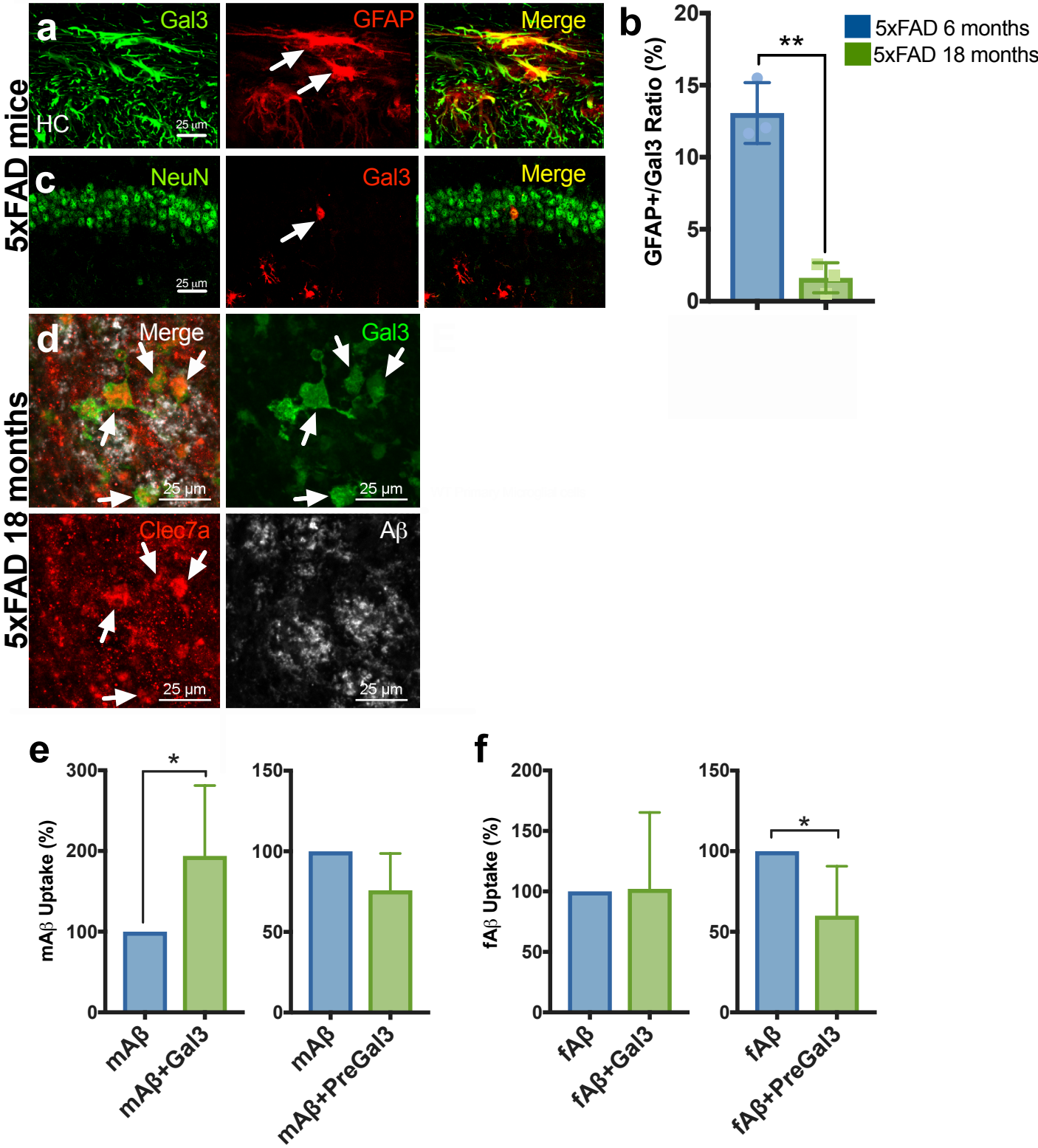

Supplement: Supplementary file 2 — Suppl. Figure 2 Rare galectin-3 protein expression in non-microglial cells. a Colocalization of GFAP (red) and galectin-3 (gal3) (green) in the hippocampal molecular layer in brain sections from 5xFAD mice, suggesting that astrocytes can be positive for gal3. b GFAP and gal3+ cell number decrease from 6 to 18 months in 5xFAD mice. c Colocalization of NeuN (green) and gal3 (red) in the pyramidal cell layer of the hippocampus in 5xFAD mouse brain sections suggests that some neurons can be positive for gal3. d Clec7a (red) and gal3 (green) colocalize in 5xFAD mice at 18 months. White arrow points to Clec7a-Gal3 colocalization. e Primary microglia treated with w/o gal3 (1 µM) before (30 min) or simultaneous with mΑβ (200 nM). f Primary microglia treated with w/o gal3 (1 µM) before (30 min) or simultaneous with fΑβ (200 nM). mΑβ, monomeric Αβ; fΑβ fibrillar Αβ (Aβ1-42 tagged with Fluor 647). Values expressed in % mAβ or fAβ uptake (vs. control) (n = 4) Statistical significance was calculated by one-way ANOVA with Tukey’s post hoc test **p < 0.01. HC = Hippocampus. Data shown as in mean ± SD (PDF 3861 kb) [file 401_2019_2013_MOESM2_ESM.pdf]

# Suppl. Fig. 3

**a**

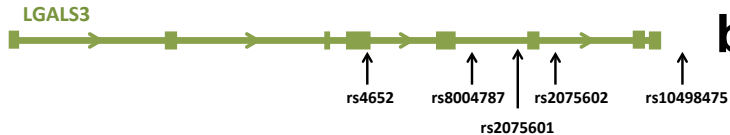

**b**

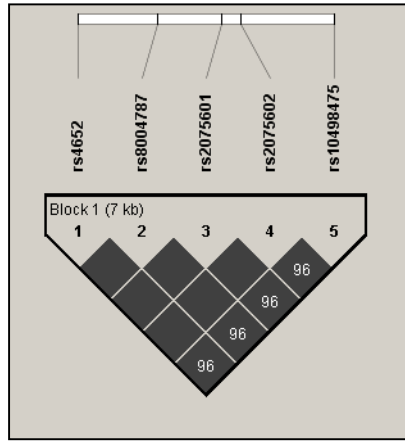

Supplement: Supplementary file 3 — Suppl. Figure 3 Single nucleotide polymorphisms associated with the gene for galectin-3 (LGALS3). a A total of 60 SNPs (single nucleotide polymorphisms) were identified in the LGALS3 genetic region. Only five SNPs were genotyped in at least three GWAS (suppl. Table 3, online resource 10). Five SNPs within the LGALS3 gene (including 1,000 bp upstream and downstream of the genetic region) were studied in relation to AD frequency in five different AD cohorts (Murcia, ADNI, GenADA, NIA and TGEN), including a total of 2,252 AD cases and 2,538 controls for the meta-analysis. b All the five studied SNPs were in high linkage disequilibrium (d´ > 0.96), suggesting non-random association of the studied alleles (PDF 160 kb) [file 401_2019_2013_MOESM3_ESM.pdf]

## Suppl. Fig. 4

## Main genes affected by the lack of gal3 in 5xFAD

**a**

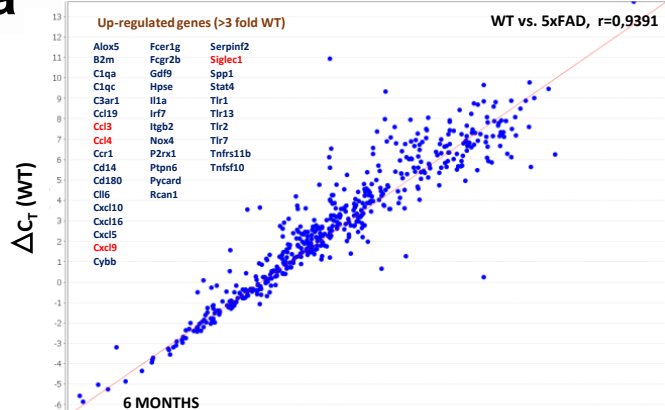

**C**

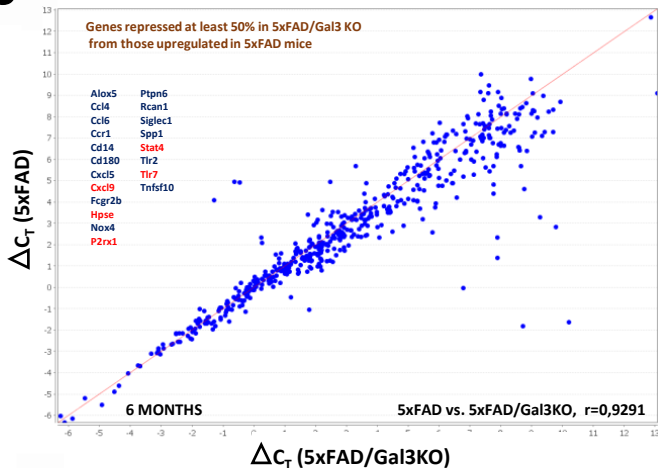

b

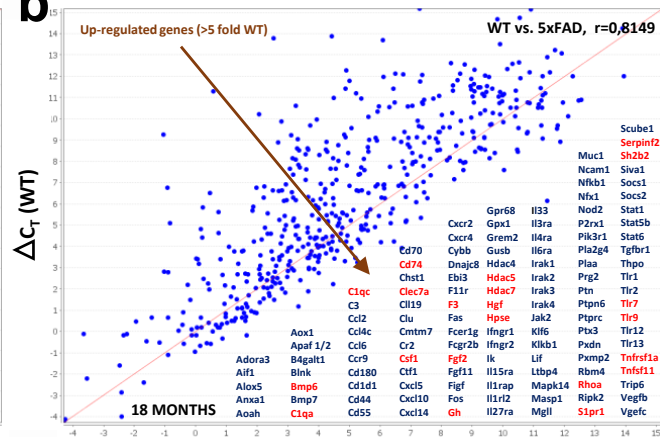

**d**

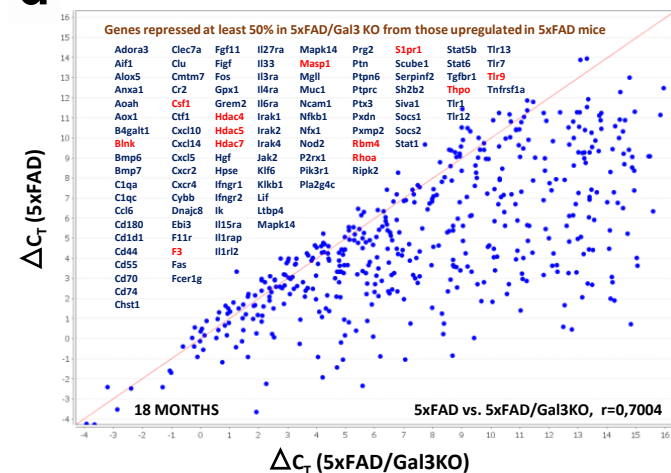

Supplement: Supplementary file 4 — Suppl. Figure 4 Inflammatory genes exhibiting the most altered expression in 5xFAD/Gal3KO mice compared to 5xFAD mice. a Upregulated genes in the hippocampi of 5xFAD mice compared to WT (>3 folds ΔCT) at 6 months old. b Upregulated genes in the hippocampi of 5xFAD mice compared to WT (>5 folds ΔCT) at 18 months old. c Downregulated genes in 5xFAD/Gal3KO mice compared to 5xFAD mice (50% cut-off of value of repressed genes) at 6 months. d Downregulated genes in 5xFAD/Gal3KO mice compared to 5xFAD mice (50% cut-off of value of repressed genes) at 18 months. Genes in red are specifically related to TLR- and TREM2-signaling (PDF 890 kb) [file 401_2019_2013_MOESM4_ESM.pdf]

# Suppl. Fig. 5

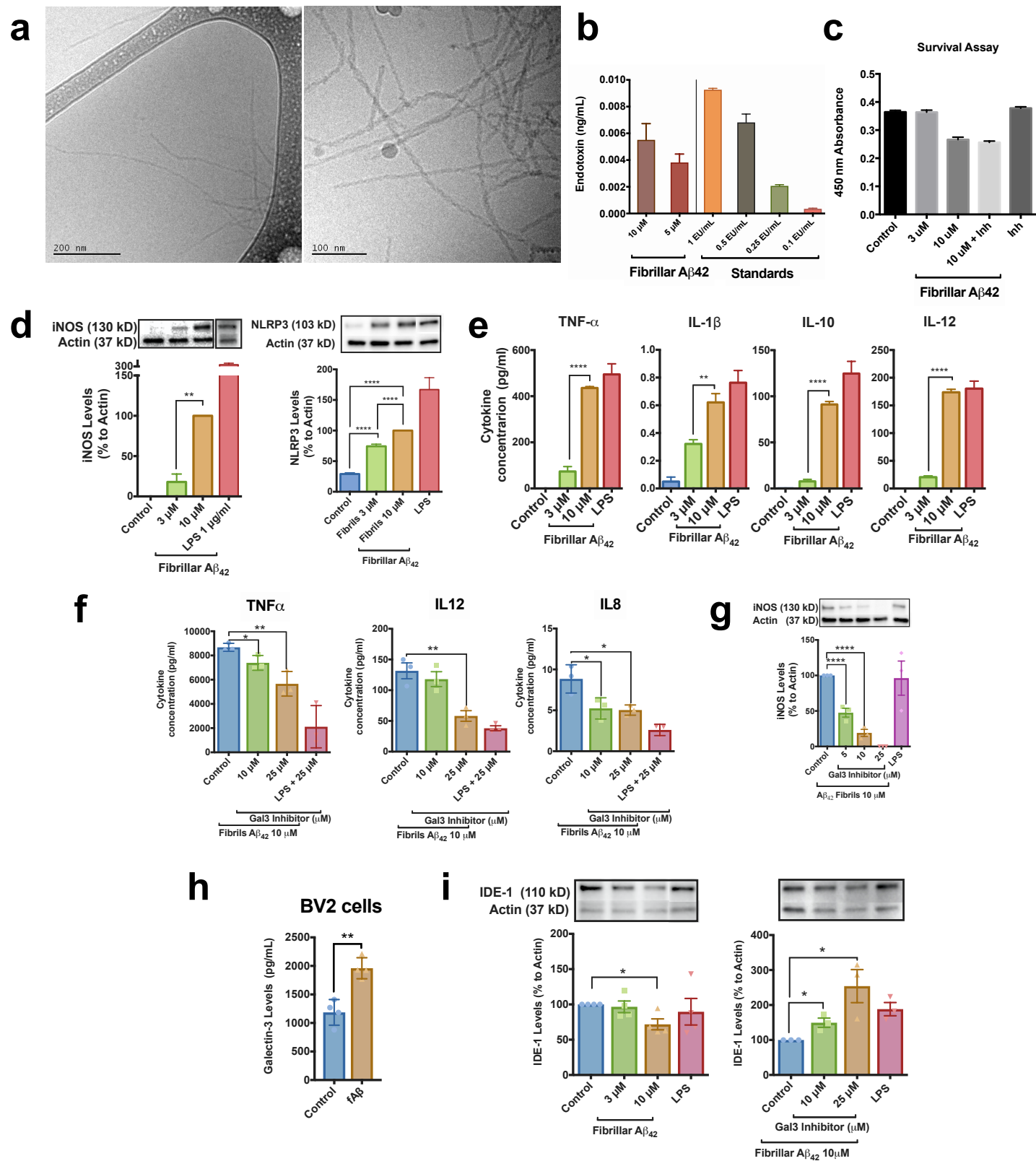

Supplement: Supplementary file 5 — Suppl. Figure 5 Characterization of the fibrils used and the activated microglia. a Electron transmission microscopy pictures of Αβ fibrils used to activate microglial cells in vitro following fibril generation. b Endotoxin assay used to measure the endotoxin levels in our Αβ fibril preparations. The levels measured were within the standards (ng/mL) and unlikely to affect the experiments. c Cell viability assay used to test cell viability following a challenge with the Αβ fibril preparation ± the gal-3 inhibitor used in our in vitro experiments. Values are expressed in absorbance 450 nm as mitochondrial activity. d iNOS levels in BV2 cells challenged with Αβ (3 and 10 μΜ) for 12 h (left). NLRP3 levels in BV2 cells challenged with Αβ (3 and 10 μΜ) for 12 h (right). LPS (1 μg/ml) was used as a control. Proteins levels are shown relative to actin levels (right). e Cytokines released into the medium by BV2 cells challenged with Αβ (3 and 10 μΜ) for 12 h. LPS (1 μg/mL) was used as a positive control. f Reduced cytokine levels culture medium in microglial BV2 cell cultures challenged with galectin-3 (gal3) inhibitor and fΑβ (fΑβ, 10 μΜ) for 12 h. g Reduced iNOS levels in BV2 cells treated with gal3 inhibitor together with fΑβ 10 μΜ for 12 h. h BV2 microglial cells increase gal3 release (culture medium) following stimulation with fΑβ. i IDE-1 levels in BV2 cells challenged with fΑβ was reduced (left, 10 μΜ), but increased when adding gal3 inhibitor (10 and 25 µM) along with fΑβ (10 μΜ) for 12h (right). In vitro experiments represent a minimum of 3 independent experiments. Statistical analysis was done using one-way ANOVA with Bonferroni’s post hoc test. **p < 0.01; ***p < 0.001; ****p < 0.0001. Data are shown as mean ± SD (PDF 2900 kb) [file 401_2019_2013_MOESM5_ESM.pdf]

# Suppl. Fig. 6

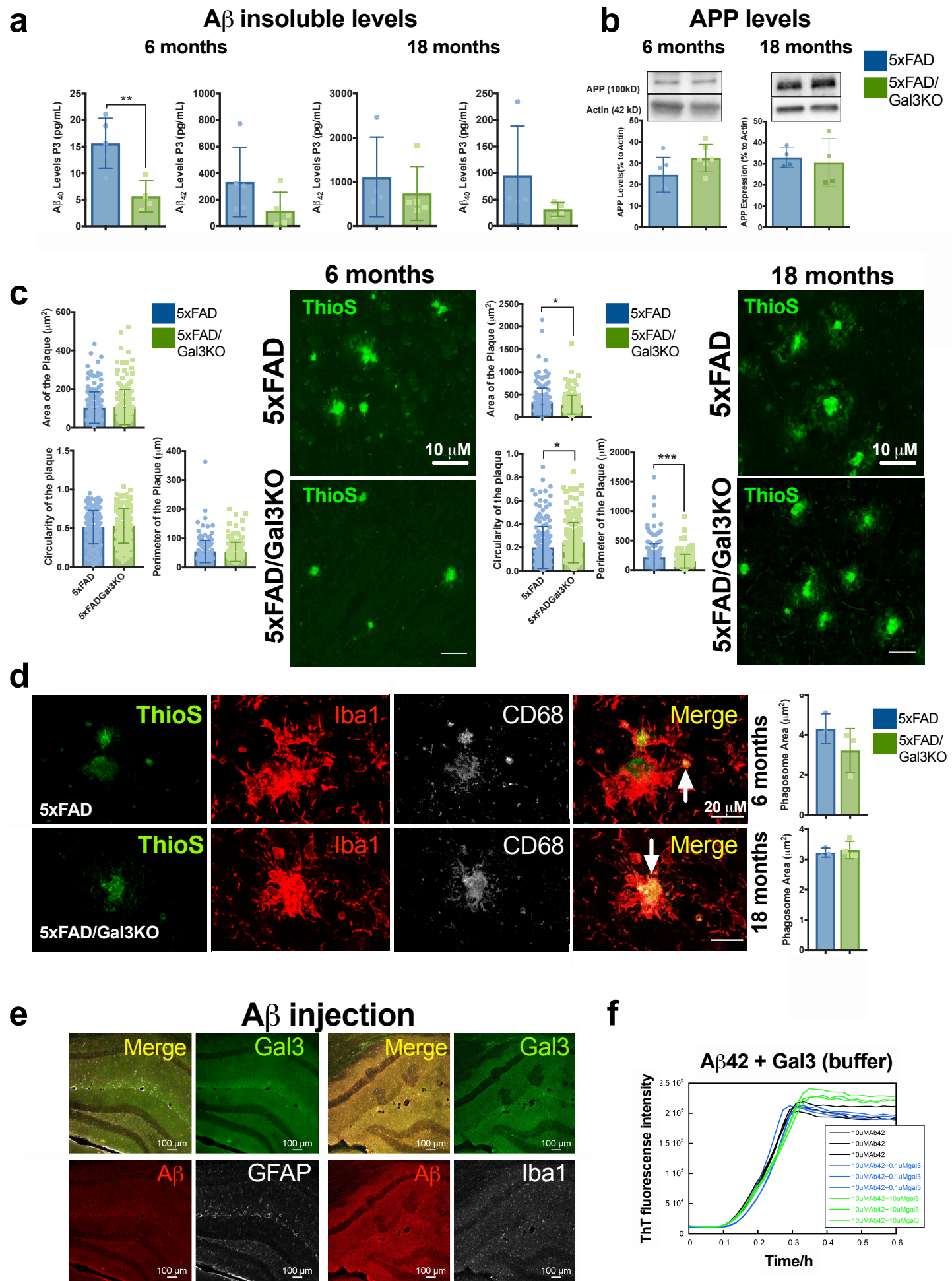

Supplement: Supplementary file 6 — Suppl. Figure 6 Evaluation of insoluble Aβ levels, Aβ plaque morphology, phagosome formation and glial reaction after Aβ injections in WT mice. a Insoluble fraction (P3) levels. Aβ42 and Aβ40 levels were measured by ELISA. The P3 fraction was extracted from the cortex of 5xFAD and 5xFAD/Gal3KO mice at 6 and 18 months. Proteins levels are in ng/mL. b APP levels were measured by western blot at 6 and 18 months in 5xFAD and 5xFAD/Gal3KO mice. c Amyloid plaque deposit analyzed at 6 and 18 months in 5xFAD and 5xFAD/Gal3KO mice (perimeter, circularity and area of the plaque). d CD68 staining performed at 6 and 18 months in 5xFAD and 5xFAD/Gal3KO mice. Phagosome area in μm2 right (white arrows point to the phagosomes). e GFAP and Iba1 immunoreactivity to Αβ injected in the hippocampi of WT mice. f ThT assay performed to characterize Αβ monomers and Αβ monomers + galectin-3 preparations injected in WT mice. Statistical analysis was performed using Student’s t-test. p** < 0.01, p*** < 0.001. Data are shown as mean ± SD (PDF 10837 kb) [file 401_2019_2013_MOESM6_ESM.pdf]

# Suppl. Fig. 7

**a**

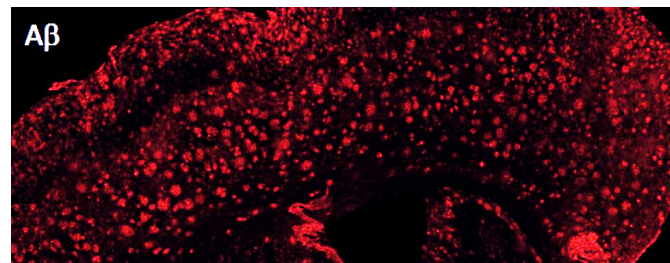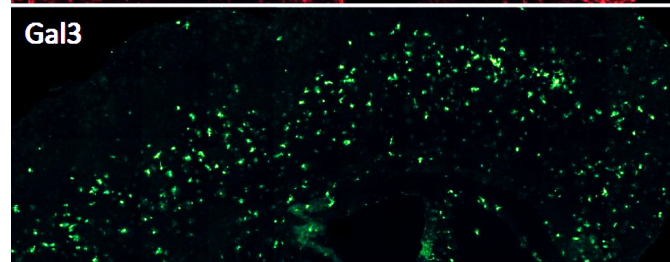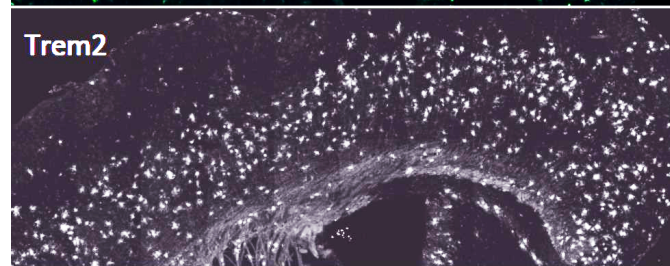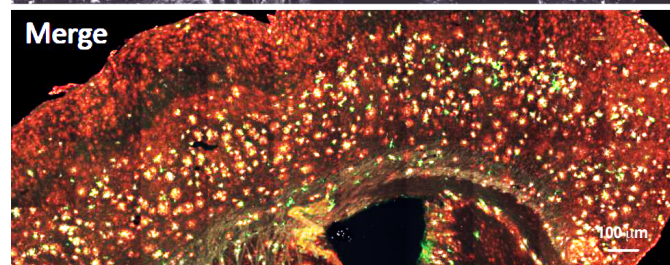

**b**

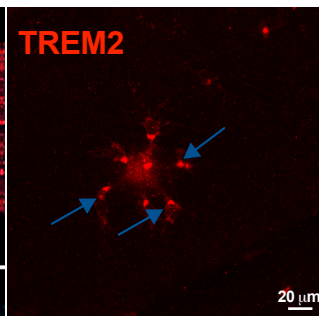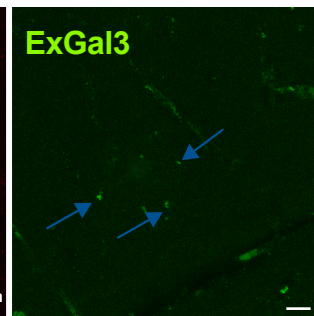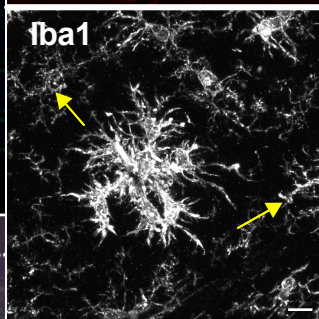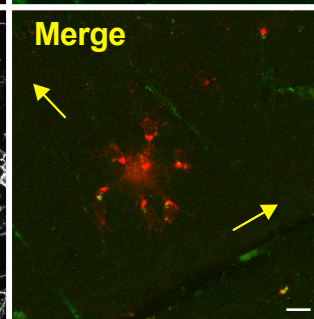

**c**

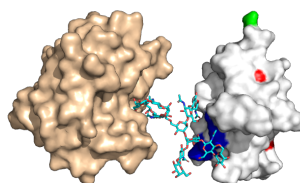

**Galectin-3 TREM-2  
Interaction**

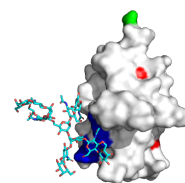

**TREM-2**

Supplement: Supplementary file 7 — Suppl. Figure 7 Galectin-3 binds to TREM2 in a CRD dependent-fashion. a Galectin-3 (gal3), Aβ and TREM2 colocalized in brain slides from 18-month-old 5xFAD mice. b Exogenous galectin-3 (ExGal3) added to 5xFAD/Gal3KO brain sections preferentially labeled TREM2-enriched areas within plaque-associated microglia. The yellow arrows point to Iba1+ microglia not to upregulating TREM2. The blue arrows point to a TREM2-gal3 (yellow) interaction in a reactive Iba1+ microglial cell exhibiting morphological features of plaque-associated microglia. c Model of TREM2 (white) with tetrantennary N-glycan (stick model) and gal3 CRD (yellow). Mutations in TREM2 associated with increased risk for AD are blue, and mutations known to cause Nasu-Hakola disease (NHD) are red. The C-terminus of the fragment, which normally links further to the transmembrane domain, is green (PDF 4440 kb) [file 401_2019_2013_MOESM7_ESM.pdf]
