# Supplementary material for: Galectin-3, a novel endogenous TREM2 ligand, detrimentally regulates inflammatory response in Alzheimer’s disease
Source: Acta Neuropathol. 2019 Apr 20;138(2):251–73. doi: 10.1007/s00401-019-02013-z (PMC6660511; doi:10.1007/s00401-019-02013-z)
Supplement: Supplementary file 8 — Supplementary material 1 (PDF 70 kb) [file 401_2019_2013_MOESM8_ESM.pdf]

Table 1. **AD subjects and healthy controls used to evaluate the role of galectin-3 in Alzheimer's disease.**

Case	***Sex***	***Age (years)***	***Post Mortem Time***	***Cause of Death***	***CNS Diagnose***
Healthy Control 1	Male	80	5h 25min	Euthanasia (colon carcinoma)	-
Healthy Control 2	Female	68	6h 50min	Euthanasia (metastatic ovarian cancer and ileus)	-
Healthy Control 3	Male	65	9h 10min	Euthanasia (heart failure)	-
AD Patient 1	Female	64	5h 15 min	Pneumonia and stomach bleeding	Alzheimer's Disease
AD Patient 2	Male	64	8h 15 min	Euthanasia (AD with Lewy's bodies)	Alzheimer's Disease with Lewis Bodies
AD Patient 3	Female	72	4h 25 min	Advanced Alzheimer's with delirium	Advanced Alzheimer's with delirium
AD Patient 4	Male	64	6h 40 min	Dehydration after CVA and AD	Alzheimer's Disease

Patients and healthy controls used to evaluate the presence of Gal3 in the vicinity of the senile plaques by immunohistochemistry.
