# Supplementary material for: Galectin-3, a novel endogenous TREM2 ligand, detrimentally regulates inflammatory response in Alzheimer’s disease
Source: Acta Neuropathol. 2019 Apr 20;138(2):251–73. doi: 10.1007/s00401-019-02013-z (PMC6660511; doi:10.1007/s00401-019-02013-z)
Supplement: Supplementary file 9 — Supplementary material 1 (PDF 96 kb) [file 401_2019_2013_MOESM9_ESM.pdf]

Table 2. **AD subjects and healthy controls used to evaluate the role of galectin-3 in Alzheimer's disease.**

Case	***Sex***	***Age (years)***	***Post Mortem Time***	***Cause of Death***	***CNS Diagnose***
Healthy Control 1	Male	80	72h	AMI	-
Healthy Control 2	Male	68	24h	Cardiac Insuff	-
Healthy Control 3	Female	65	24h	AMI	-
Healthy Control 4	Female	86	4 days	Aorta-diss. O rupture	-
Healthy Control 5	Male	75	72h	AMI	-
AD patient 1	Male	77	48h	Pneumonia	Alzheimer's Disease-FTD
AD Patient 2	Male	82	7 days	AMI+Septicemia	Alzheimer's Disease
AD Patient 3	Male	63	96h	Pulm Embolus	Alzheimer's Disease
AD Patient 4	Male	76	48h	Pneumonia + AMI	Alzheimer's Disease
AD Patient 5	Male	80	48h	Pneumonia + Car.Insuff	Alzheimer's Disease
AD Patient 6	Female	70	4 days	Pneumonia + AMI	Alzheimer's Disease

*AMI = *Acute Myocardial Infarction*. Patients and healthy controls were used to evaluate the inflammatory component (IL6), the levels of Gal3 and amyloid beta by western blot and ELISA.
