# Supplementary material for: Galectin-3, a novel endogenous TREM2 ligand, detrimentally regulates inflammatory response in Alzheimer’s disease
Source: Acta Neuropathol. 2019 Apr 20;138(2):251–73. doi: 10.1007/s00401-019-02013-z (PMC6660511; doi:10.1007/s00401-019-02013-z)
Supplement: Supplementary file 10 — Supplementary material 1 (PDF 136 kb) [file 401_2019_2013_MOESM10_ESM.pdf]

CHR	BP	SNP	A1	A2	N	P	P(R)	OR	OR(R)	Q	I	ADNI	INIA	GENADA	MUR
14	54674789	rs4652	C	A	3	0.022	0.022	1.11	1.11	0.4455	0.00	1.33	1.13	1.07	NI
14	54677119	rs8004787	T	C	3	0.027	0.023	1.10	1.11	0.4418	0.00	1.33	1.10	1.07	NI
14	54678987	rs2075601	T	C	3	0.022	0.022	1.11	1.11	0.4455	0.00	1.33	1.11	1.07	NI
14	54679527	rs2075602	G	A	3	0.027	0.023	1.10	1.11	0.4418	0.00	1.33	1.10	1.07	NI
14	54682233	rs10498475	T	C	3	0.018	0.018	1.27	1.23	0.4896	0.00	0.96	1.25	NI	1.51

CHR, chromosome; BP, base pair position; SNP, single nucleotide polymorphism; A1, reference allele; A2, alternative allele; N, number of GWAS included in the meta-analysis; p, fixed-effects p-value; p (R), random-effects p-value; OR, fixed-effects odds ratio; OR (R), random-effects odds ratio; Q, p-value for heterogeneity of OR; I, effect size for heterogeneity of OR. The last four columns show the OR for each GWAS.
